# Exploration of the Phytochemical and Antidiabetic Properties of *Teucrium polium*: A Natural Asset for Type 2 Diabetes Management

**DOI:** 10.1002/open.202500346

**Published:** 2025-11-18

**Authors:** Hajar El Ouadni, Aziz Drioiche, Fadoua El Makhoukhi, Omkulthom Al kamaly, Hannou Zerkani, Smail Amalich, Imane Tagnaout, Mohamed Radi, Yahya Cherrah, Touriya Zair, Katim Alaoui

**Affiliations:** ^1^ Research Team on the Toxicity and Pharmacodynamics of Aromatic and Medicinal Plants (ERTP.PAM) Pharmacology and Toxicology Laboratory Rabat Faculty of Medicine and Pharmacy (FMPR) Mohammed V University (UMV) Rabat B.P., 6203 Morocco; ^2^ Research Team of Chemistry of Bioactive Molecules and the Environment Laboratory of Innovative Materials and Biotechnology of Natural Resources Faculty of Sciences Moulay Ismaïl University B.P., 11201, Zitoune Meknes 50070 Morocco; ^3^ Higher Institute of Nursing Professions and Health Techniques of Fez Regional Health Fez‐Meknes EL Ghassani Hospital Fez 30050 Morocco; ^4^ Laboratory of Spectroscopy, Molecular Modelling, Materials, Nanomaterial, Water and Environment, CERNE2D Faculty of Science Mohammed V University in Rabat Rabat 10100 Morocco; ^5^ Department of Pharmaceutical Sciences College of Pharmacy Princess Nourah bint Abdulrahman University P.O., Box, 84428 Riyadh 11671 Saudi Arabia; ^6^ Laboratory of Phytochemistry National Agency of Medicinal and Aromatic Plants (ANPMA) BP:, 159, Taounate Principale Taounate 34000 Morocco

**Keywords:** antidiabetic, antimicrobial, antioxidant, apigenin‐7‐rutinoside, carvacrol, poliumoside, *teucrium polium*, verbascoside

## Abstract

Type 2 diabetes (T2D) is characterized by hyperglycemia due to impaired insulin utilization, and current therapies face notable limitations. This study investigated the chemical composition and biological activities of *Teucrium polium* essential oils and extracts, with a focus on their antidiabetic, antimicrobial, and antioxidant properties. Essential oil from aerial parts (yield 1.65%) was obtained by hydrodistillation; extracts were prepared by aqueous decoction (E0) and Soxhlet (aqueous, E1; hydroethanolic, E2). HPLC‐UV‐Vis‐ESI‐MS and GC–MS identified bioactives. The oil was dominated by carvacrol (28.10%), thymol (26.28%), *γ*‐terpinene (12.11%), and o‐cymene (15.59%). E0 was rich in poliumoside (36.45%); E1 contained verbascoside (9.42%) and isorhamnetin‐3‐O‐rutinoside (9.68%); E2 was dominated by apigenin‐7‐rutinoside (21.18%). Antioxidant assays showed 85% DPPH inhibition at 100 µgmL^–^^1^, FRAP EC_50_ of 25.4 µgmL^–1^, and 75% TAC inhibition at 100 µg mL^–1^. Antimicrobial activity yielded MICs of 0.5 mg mL^–1^ for *Staphylococcus aureus* and *Escherichia coli* and 0.3 mg mL^–1^ for *Candida albicans*. Antidiabetic assays demonstrated 65% inhibition of *α*‐amylase and 72% inhibition of *α*‐glucosidase at 100 g mL^–1^. In vivo, glucose tolerance testing showed a 30% reduction in postprandial glycemia at 70 mg kg^–1^ and near‐normal glycemia after 7 days. These findings support *T. polium*'s traditional use for T2D and warrant further toxicological and clinical evaluation.

## Graphical abstract

Teucrium polium shows antidiabetic activity with its phytochemical composition and biological activities. The essential oil is rich in carvacrol, thymol, *γ*‐terpinene, and o‐cymene, while the aqueous and hydroethanolic extracts are characterized by poliumoside, verbascoside, isorhamnetin‐3‐O‐rutinoside, and apigenin‐7‐rutinoside. Antioxidant activity is strong (85% DPPH, FRAP EC50 25.4 μg mL^–1^), with significant antimicrobial activity (MIC down to 0.3 mg mL^–1^) and strong inhibition of digestive enzymes (65% *α*‐amylase, 72% *α*‐glucosidase). In vivo, glucose tolerance is enhanced, with a 30% decrease in postprandial hyperglycemia at 70 mg kg^–1^ and near‐normal glycemia after 7 days. These results validate the traditional use of T. polium and warrant further toxicological and clinical studies.

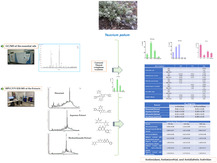

## 1 Introduction

Persistent hyperglycemia resulting from insulin resistance and/or insufficient insulin secretion by pancreatic *β*‐cells is a hallmark of type 2 diabetes (T2D), a chronic metabolic disorder.^[^ ^1^ ^]^ Due to its rising prevalence and related complications, such as neuropathies, diabetic nephropathy, and cardiovascular diseases, this disease poses a serious threat to global public health.^[^ ^2^ ^]^ Despite being widely prescribed, conventional treatments like metformin and DPP‐4 inhibitors have drawbacks, such as unfavorable side effects, ineffectiveness in certain patients, and the potential to develop long‐term resistance.^[^ ^3^ ^]^ In this regard, medicinal plants offer a promising therapeutic option for treating T2D. Traditional medicine has utilized a variety of plant species for centuries to regulate blood sugar levels and prevent complications associated with this condition.^[^ ^4^ ^]^ By enhancing insulin sensitivity, blocking glucose‐digesting enzymes, and shielding pancreatic cells from oxidative stress, several plants, including *Momordica charantia*, *Cinnamomum verum*, and *Trigonella foenum‐graecum*, have demonstrated hypoglycemic effects.^[^ ^5^ ^]^ *T. polium* L., a perennial plant from the Lamiaceae family that grows in the Mediterranean and dry areas of the Middle East and North Africa, is known for having a wide range of pharmacological effects, such as being an antioxidant, anti‐inflammatory, and antidiabetic.^[^ ^6^ ^]^ It has been long used in traditional medicine, particularly for treating infections, metabolic diseases, and gastrointestinal issues.^[^ ^7^ ^]^ Its abundance in bioactive substances, such as terpenoids, phenolic acids, and flavonoids (including quercetin and luteolin), is thought to modulate glucose metabolism by promoting glucose uptake in peripheral tissues and controlling insulin signaling.^[^ ^8^ ^]^

The Lamiaceae family includes the perennial herb *T. polium* L., which is also known as woolly germander or Polium germander. This species is found all over the Mediterranean Basin, the Middle East, and North Africa. It performs best in dry and semi‐dry environments, where it often settles on rocky, nutrient‐poor soils.^[^ ^9^ ^]^ *T. polium* has gray‐green leaves and flowers that grow in clusters. The flowers are small, tubular, and can be white or purple, depending on the subspecies and weather conditions.^[^ ^10^ ^]^ The plant is widely used in traditional medicine, particularly in Mediterranean, Persian, and Arab pharmacopoeias, where it has been employed for hundreds of years to treat a range of ailments, including digestive problems, type 2 diabetes, inflammatory diseases, and general fatigue.^[^ ^11^ ^]^ Ancient texts, such as those by Dioscorides and Avicenna, document its historical use, indicating a long‐standing tradition of medicinal application.^[^ ^12^ ^]^

*T. polium* L.'s pharmacological effects come from its wide range of phytochemicals. The plant is a rich source of bioactive compounds, such as flavonoids (like apigenin, luteolin, and quercetin), terpenoids (especially diterpenes like teucine A and poliumoside), phenolic acids (like rosmarinic acid and caffeic acid), iridoids (like harpagoside and aucubine), and essential oils (EO).^[^ ^13^ ^,^ ^14^ ^]^ Flavonoids are recognized for their potent antioxidant and anti‐inflammatory effects, while iridoids possess properties that protect the liver, immune system, and brain.^[^ ^15^ ^]^ Terpenoids, particularly monoterpenes and diterpenes, are gaining increasing attention because they may be able to combat cancer, bacteria, and inflammation.^[^ ^16^ ^]^ The composition of *T. polium* EOs can vary significantly depending on where they are grown, the type of soil they are cultivated in, and the prevailing weather conditions. Still, they usually contain bioactive compounds such as *β*‐pinene, linalool, germacrene‐D, and *α*‐terpineol, which help them fight germs and free radicals.^[^ ^17^ ^]^

Over the last few decades, rigorous in vitro and in vivo studies have demonstrated that *T. polium* exhibits considerable pharmacological potential. The plant exhibits strong antioxidant properties, primarily through the neutralization of free radicals, binding of metal ions, and induction of endogenous antioxidant enzyme expression.^[^ ^18^ ^]^ Due to these traits, *T. polium* is a promising candidate for treating diseases associated with oxidative stress, such as diabetes, cardiovascular diseases, and neurodegenerative disorders.^[^ ^19^ ^]^ Researchers have shown that *T. polium* extracts can stop the production of pro‐inflammatory cytokines (like IL‐1*β*, TNF‐*α*, and IL‐6) and stop the activation of NF‐*κ*B and MAPK signaling pathways in different experimental models.^[^ ^20^ ^]^ These effects suggest that they could be beneficial in treating chronic inflammatory diseases, such as rheumatoid arthritis, inflammatory bowel diseases, and metabolic syndrome.^[^ ^20^ ^]^

Another critical area of research is *the ability of Teucrium polium* to inhibit the growth of germs. Studies have shown that its extracts and EOs are effective against a wide range of bacterial and fungal pathogens, including *Staphylococcus aureus*, *Escherichia coli*, *Candida albicans*, and *Aspergillus niger*.^[^ ^21^ ^]^ This antimicrobial effectiveness, combined with its long history of use in treating infections, suggests that it could be a source of new antimicrobial agents, particularly in areas where access to regular antibiotics is limited.^[^ ^22^ ^]^ *T. polium* has been shown to have promising antidiabetic effects in addition to its antioxidant, anti‐inflammatory, and antimicrobial properties. Studies have shown that its extracts alter the amount of insulin released, increase the uptake of glucose by peripheral tissues, and inhibit key enzymes involved in carbohydrate metabolism, such as *α*‐amylase and *α*‐glucosidase.^[^ ^8^ ^]^ It appears that flavonoids and terpenoids collaborate to enhance insulin sensitivity and reduce high blood sugar levels in animal models of type 2 diabetes.^[^ ^23^ ^]^ Additionally, early research indicates that *T. polium* may induce apoptosis in specific cancer cell lines, including those associated with breast, colon, and liver cancers, by inhibiting the cell cycle and blocking angiogenesis.^[^ ^24^ ^]^

Although *T. polium* has some pharmacological potential, its safety profile requires further examination. Some studies have found that using *T. polium* extracts for an extended period or in high doses can be harmful to the liver, especially those containing a high concentration of diterpenes, such as teucine A. These diterpenes can damage the liver by inducing oxidative stress and disrupting mitochondrial function.^[^ ^25^ ^]^ These results demonstrate the importance of conducting thorough toxicological evaluations and establishing precise dosing guidelines to minimize potential risks.^[^ ^26^ ^]^ From an ecological perspective, *T. polium* is highly significant in Mediterranean and dry ecosystems, where it grows naturally. It helps keep these delicate ecosystems stable, prevents erosion, and maintains biodiversity by growing in soils that lack nutrients.^[^ ^27^ ^]^ However, the growing demand for *T. polium* as a medicinal plant has led to overexploitation and degradation of its habitat, which threatens its wild populations.^[^ ^28^ ^]^ For this valuable plant to survive in the long term, both for its medicinal and ecological benefits, it is important to use sustainable farming methods and conservation strategies.^[^ ^29^ ^]^

The primary objective of this study is to thoroughly investigate the chemical composition and biological activities of the EO and extracts of *Teucrium polium*, with a focus on their antibacterial, antifungal, antioxidant, and antidiabetic properties. Advanced phytochemical profiling techniques, such as gas chromatography‐mass spectrometry (GC–MS) and high‐performance liquid chromatography (HPLC), coupled with ultraviolet–visible (UV) and electrospray ionization (ESI)‐mass spectrometry (MS), will be used to identify and quantify the bioactive constituents. In vitro tests will assess the effectiveness of the EO and extracts of *T. polium* against microbial pathogens, oxidative stress, and the modulation of glucose metabolism. By integrating phytochemical and biological analyses, this research aims to validate the traditional uses of *T. polium* EO and extracts, identify promising candidates for therapeutic development, and expand the knowledge of this pharmacologically significant plant.

## 2 Experimental Section

### 2.1 Collection and Identification of Plant Material

The aerial parts of *T. polium* were collected in the spring of 2024 from the Rommani region, located in the Moroccan Atlas Mountains (33°30’11.1"N 6°36’05.4" W), selected for its ecological significance and the natural abundance of the species. The taxonomic identification was confirmed by Professor Amina Bari from the Department of Botany at the Faculty of Sciences in Fès. After collection, the plant material was dried at room temperature, away from light, for 2 weeks to preserve the integrity of its bioactive metabolites. Once dried, a portion of the sample was ground for decoctions and Soxhlet extraction. In contrast, the remaining portion was cut into small pieces and stored in airtight containers, protected from moisture and light, until it was used for experimental analyses.

### 2.2 Microbial Material

We tested the extracts and EO of *T. polium* against three types of bacteria (*Escherichia coli*, *Staphylococcus aureus*, and *Klebsiella pneumoniae*) and three types of fungi (*Candida albicans*, *Aspergillus niger*, and *Saccharomyces cerevisiae*). These microorganisms were isolated from clinical samples collected from patients at the Ibn Sina Hospital Centre in Morocco. The strains were initially preserved in a 20% glycerol solution at −80 °C and then reactivated in Mueller–Hinton broth for the bacteria, Sabouraud dextrose broth for *Candida albicans* and *Aspergillus niger*, and YPD (Yeast Peptone Dextrose) broth for *Saccharomyces cerevisiae* before being cultured.

### 2.3 Quality Control of Plant Material

#### 2.3.1 Determination of pH

The acidity of the product is quantitatively expressed by its pH value. The analytical procedure involves dissolving 2.0 g of the sample in 10 mL of hot distilled water, followed by cooling, filtration, and further cooling of the resulting mixture. The pH measurement is then performed by immersing a previously calibrated electrode into a sufficient volume of the filtrate to obtain accurate and stable readings.^[^ ^30^ ^,^ ^31^ ^]^

#### 2.3.2 Mineral Content (Ash) and Organic Matter Content

The determination of mineral content is based on the calcination of the plant sample. For this purpose, 2.0 g of finely ground material is placed in a preheated muffle furnace at 550 °C. The sample is heated until the complete volatilization of carbon residues, leaving only a homogeneous ash residue with constant weight.^[^ ^32^ ^]^ This procedure ensures the destruction of organic compounds, and the obtained mineral fraction allows for the calculation of the organic matter content using the following Formula (1):

(1) $$\mathrm{OM}\%=\left(\frac{m_{1}-m_{2}}{\text{PE}}\right)\times 100$$

where OM% represents the organic matter content, *m*_1_ is the mass of the capsule and sample before calcination, *m*_2_ is the mass of the capsule and sample after calcination, PE is the mass of the analyzed sample. The ash content is calculated using the following Equation (2)

(2) Ash%=100−OM%

#### 2.3.3 Moisture Content (MC)

The determination of water content follows the AFNOR standard (NF‐V03‐402, 1985).^[^ ^33^ ^]^ A 5 g sample is subjected to drying at 103 °C for 24 h and then cooled in a desiccator before being reweighed. The mass loss recorded allows for the calculation of the moisture content, expressed as a percentage of the dry matter, using the following Formula (Formula 3)

(3) $$\text{MC}\%=\left(\frac{m_{0}-m_{1}}{m_{0}}\right)\times 100$$

where *m*_0_ is the initial mass of the sample, *m*_1_ is the mass of the sample after drying.

#### 2.3.4 Acidity

To quantify the acidity, 10.0 g of finely ground plant material was mixed with 100 mL of boiling distilled water and continuously stirred for 15  min to ensure adequate extraction of the organic acids. The resulting mixture was then filtered to separate the liquid phase. Ten milliliters of the filtrate was transferred to a beaker, diluted with 20 mL of distilled water, and a few drops of phenolphthalein indicator were added. The sample was titrated with a 0.01 N NaOH solution until the endpoint was reached, indicated by a persistent pink color. The volume of NaOH consumed was recorded, and the acidity was calculated in citric acid equivalents using the following Formula (4)^[^ ^34^ ^]^

(4) $$\text{Acidity}\left(\%\right)=\frac{\text{dilution factor}*\text{acid equivalent weight}*\text{normality of NaOH}*\text{titration volume}\left(\mathrm{ml}\right)}{\text{weight of sample}\;(g)}$$

The final result was expressed as a percentage of citric acid, calculated based on the equivalent weight of citric acid.

#### 2.3.5 Determination of Mineral Composition by ICP‐AES

The content of trace metals, heavy metals, and trace elements was determined by inductively coupled plasma atomic emission spectroscopy (ICP‐AES). This method enables the rapid and accurate quantification of both minor and major mineral components. One gram of finely powdered plant material was subjected to mineralization by heating with a mixture of 5 mL of concentrated nitric acid (HNO_3_) and 15 mL of hydrochloric acid (HCl) at 110 °C. The sample was fully broken down and dissolved and then cooled to room temperature and mixed with ultrapure water to make a final volume of 100 mL. The UATRS (Technical Support Unit for Scientific Research) laboratory tested the resulting solution twice, using an Agilent 5110 ICP‐AES spectrometer to directly measure the concentrations of trace metals. The concentrations of trace metals were determined and expressed in milligrams per liter (mg L^–^^1^).^[^ ^35^ ^]^

### 2.4 Phytochemical Screening

Phytochemical screening is a qualitative method used to identify the chemical families present in a plant. This approach relies on reactions that either generate insoluble complexes through precipitation or produce colored complexes through coloration.^[^ ^36^, ^37^, ^38^, ^39^ ^–^ ^40^ ^]^

### 2.5 Extraction of the EO

The EO was extracted from the plant material by hydrodistillation. One hundred grams of dried flowering tops were mixed with 1 L of distilled water and heated at 90 °C for 3 h in a Clevenger apparatus. The volatile compounds were carried by steam through the condenser, where the mixture was cooled to form a biphasic liquid phase. The EO, which has a lower density than water, was separated and collected. It was then dried over anhydrous sodium sulfate and stored in an airtight brown glass bottle at 4 °C to prevent degradation.

### 2.6 Essential Oil Yield

The EO yield (%EO) is determined by expressing the volume of EO in relation to the mass of dry plant material (V m^–^^1^). The calculation is performed using the following Formula (5)

(5) $$\text{\% EO}=\frac{V}{m}\times 100$$

where *V* is the volume of the obtained EO (in mL), *m* is the mass of the dry plant material (in g). Quality control of the oil is essential to ensure the safety of products intended for human consumption.

### 2.7 Determination of Density

The density of an EO at 20 °C is determined by calculating the ratio of the oil's mass to its volume, compared to the density of pure water at the same temperature.^[^ ^41^ ^]^ The Formula (6) used to calculate the density of the EO is as follows

(6) $$d_{20}=\frac{\left(m_{2}‐m_{0}\right)}{\left(m_{1}‐m_{0}\right)}$$

where *m*_0_ is the mass in grams of the empty pycnometer, *m*_1_ is the mass in grams of the pycnometer filled with water, *m*_2_ is the mass in grams of the pycnometer filled with essential oil.

### 2.8 Analysis and Identification of EO by GC–MS

The analysis of the essential oil of *T. polium* is performed using gas chromatography coupled with mass spectrometry (GC–MS) with a Thermo Electron chromatograph (Trace GC Ultra) equipped with a DB‐5 capillary column (5% phenyl‐methyl‐siloxane, 30 m × 0.25 mm; film thickness: 0.25 μm). The injection is carried out in split mode (1/70) with a volume of 1 μL. Helium is used as the carrier gas at a flow rate of 1.5 mL min^–^^1^, and the temperature is programmed to increase at a rate of 4 °C min^–1^. The identification of the constituents is performed using a Thermo Electron Trace MS (Polaris Q) mass spectrometer, operating at an electron impact energy of 70 eV. The obtained mass spectra are compared with those from the NIST 98 library to identify the compounds present.^[^ ^42^ ^]^ The indexing of the compounds is based on the calculation of Kovats indices, established by injecting a hydrocarbon standard (C7‐C40) under the same analytical conditions. These indices are determined using the following relation (7)

(7) $$\text{KI}=\left[\frac{\left({\text{RT}}_{x}-{\text{RT}}_{n}\right)}{\left({\text{RT}}_{n+1}-RT_{n}\right)}+n\right]\times 100$$

where KI is the Kovats index, RT_*x*_ is the retention time of the target compound, RT_*n*_ and RT_*n* + 1_ are the retention times of the reference hydrocarbons with n and *n* + 1 carbon atoms, respectively, *n* corresponds to the number of carbon atoms of the eluted hydrocarbon immediately before the target compound. The calculated indices, along with the mass spectra, are compared to reference databases to ensure a rigorous identification of the essential oil constituents.

### 2.9 Extraction and Characterization of Polyphenolic Compounds from T. polium

#### 2.9.1 Extraction and Yield

The phenolic compounds from *T. polium* were extracted using two solid–liquid methods: Soxhlet extraction and decoction. Soxhlet extraction: 30 g of plant powder was extracted with 300 mL of ethanol or water for 6 h. After heating and percolation, the extracts were filtered, concentrated under reduced pressure, and then weighed to determine the yield. Decoction: 30 g of powder was boiled in 600 mL of distilled water at 80 °C for 1 h. After cooling and filtration under low pressure, the extract was dried at 70 °C and stored as a powder. The extracts were coded according to the details in **Table** **1**.

**Table 1:** Extract codes.

Extraction method	Solvents	Code
Soxhlet	Ethanol (70%)	E (2)
	Water	E (1)
Decoction	Water	E (0)

The following formula is used to calculate the extraction yields (Y) for both decoction and Soxhlet extraction (8)

(8) $$Y=\left(\frac{m_{\text{extract}}}{m_{0}}\right)*100$$

where *m*_extract_ represents the mass of the active components present in the dry material after extraction, not the total weight of the extract, focusing only on the substances that contribute to the effectiveness of the extract, *m*_0_ is the initial mass of the plant material before extraction.

#### 2.9.2 Total Polyphenolic Compounds Assay

The polyphenol content of the different plant extracts was determined using the Folin–Ciocalteu reagent, following a slightly modified version of the method described by Singleton and Rossi.^[^ ^43^ ^]^ An aliquot of 0.1 mL of each extract was mixed with 2 mL of a freshly prepared 2% sodium carbonate solution. The mixture was vortexed and incubated for 5 min. Next, 100 μL of 0.1 N Folin–Ciocalteu reagent was added, and the solution was incubated at room temperature in the dark for 30 min. The absorbance of the extracts was measured at 760 nm using a spectrophotometer, with a blank solution serving as the reference. The polyphenol content in the extracts was determined based on the absorbance at 760 nm. A calibration curve was prepared under the same conditions, using gallic acid as the standard, starting from a concentration of 45 μg mL^–^^1^ and followed by serial dilutions to construct the curve. The results were expressed as milligrams of gallic acid equivalents per gram of dry plant material (mg GAE/g). Calculations were performed using the calibration equation *Y* = 0.077X + 0.001 (*R* ^2^ = 0.996). Each experiment was conducted in triplicate.

#### 2.9.3 Flavonoid Assay

The colourimetric method with aluminum chloride (AlCl_3_), as described by Djeridane^[^ ^44^ ^]^ and Hung,^[^ ^45^ ^]^ was used to determine the amount of flavonoids in the crude extracts. In this test, yellow complexes are formed by aluminum chloride with the oxygen atoms in flavonoids at positions 4 and 5. According to Chang et al.'s protocol, 1 mL of each extract (diluted in its original solvent) was mixed with 1 mL of a methanol solution containing 2% AlCl_3_. The absorbance at 430 nm was measured using a UV‐VIS spectrophotometer after a 10‐minute reaction time, with concentrations ranging from 5 to 35 μg mL^–1^. A calibration curve (*Y* = 0.0421X – 0.0763; *R* ^2^ = 0.9512) was created using quercetin standards to measure the flavonoid content in micrograms of quercetin equivalents per milligram of dry extract (μg QE/mg extract). The experiment was repeated three times to ensure the accuracy of the results.

#### 2.9.4 Condensed Tannin Assay

The vanillin method^[^ ^46^ ^]^ was used to determine the amount of condensed tannin present. To each sample or standard (400 μL), 3 mL of a 4% vanillin solution in methanol and 1.5 mL of concentrated hydrochloric acid were added. The absorbance of the mixture was measured at 500 nm against a blank after incubation for 15 min. The concentrations of condensed tannins were determined using a catechin calibration curve (0–300 μg mL^–1^). The amount of condensed tannin present was determined using the catechin‐based calibration curve (*Y* = 0.0027X − 0.1232; *R* ^2^ = 0.9872). Results were expressed as micrograms of catechin equivalents per milligram of extract (μg ECT/mg extract).

#### 2.9.5 Determination of the Content of Polyphenolic Compounds, Flavonoids, and Tannins

Formula (9) is used to calculate, for the sample, the content (T) of each family of phenolic compounds, flavonoids, tannins, or total polyphenols, where T denotes a content or concentration expressed in the same units. This way, the measurement only shows the active phenolic components and not the whole weight of the extract

(9) $$T=\left[\frac{C.V_{\text{effective}}}{m}\right]*D$$

where *C* represents the mass concentration determined according to the calibration curve, *V*_effective_ is the volume of the extract containing only the active phenolic components (polyphenolic compounds, flavonoids, and tannins); *m* is the mass of the dry plant material used for extraction; *D* is the dilution factor, with *D* *=* *V*_f_ */ V*_i_; *V*_f_ is the final volume measured by spectrophotometry; *V*_i_ is the volume of the extract used for analysis.

#### 2.9.6 Quantitative Analysis of Phenolic Compounds by HPLC/UV‐ESI‐MS

The analysis by HPLC/UV‐ESI‐MS was performed using a system equipped with a C18 column (3 µm) to ensure optimal separation of the analytes. The mobile phase consisted of two solvents: solvent A, which was ultrapure water with 0.1% formic acid, and solvent B, which was either pure methanol or a 50:50 (v/v) mixture of methanol and acetonitrile with 0.1% formic acid to improve the solubility of the phenolic compounds. The elution followed a specific gradient, starting at 2% solvent B, increasing gradually to 20% at 10 min, 50% at 20 min, and reaching 95% at 30 min, which was maintained until 35 min before returning to 2% at 37 min with a final re‐equilibration at 40 min. The mobile phase flow rate was set to 0.5 mL min^–^^1^ to optimize interaction with the stationary phase and avoid peak broadening. The mobile phase was degassed by ultrasound before use, and a constant nitrogen flow was applied when available to minimize bubble formation. The column was maintained at 35 °C to preserve the integrity of thermosensitive analytes, though a temperature of 40 °C was used for thermally stable analytes. The injection volume was set to 10 µL to prevent overload effects and ensure optimal separation of compounds. The samples were stored at 5 °C before analysis to ensure stability.

UV spectra acquisition was performed at 280, 320, and 360 nm, wavelengths specific to phenolic compounds, with the analysis range extended between 190 and 400 nm to detect other potential absorptions. The mass spectrometry analysis was conducted in negative ionization mode (ESI‐) with a capillary voltage of 3500 V, a drying gas temperature of 220 °C, a gas flow rate of 10 L min^–^^1^, and a gas pressure of 3 bars. The spectra were acquired over a mass range from m/z 50 to 1000, enabling the detection of a wide variety of compounds, including those with high molecular masses.

### 2.10 Study of the Antioxidant Activity

#### 2.10.1 DPPH Test

The DPPH (2,2‐diphenyl‐1‐picrylhydrazyl) test is used to measure the radical‐scavenging activity of molecules or plant extracts. DPPH is a stable radical with a purple color and a maximum absorbance at 517 nm.^[^ ^47^ ^]^ To make a 2.4% DPPH solution, mix 2.4 mg of DPPH with 100 mL of ethanol and shake for 30 min. The tubes are filled with extracts, and then 200 µL of ethanol is added. After that, 2.8 mL of the DPPH solution is added, and the mixture is left to sit for 30 min. The absorbance at 517 nm was measured using a control containing ethanol and DPPH, which was maintained under the same conditions. Pure ethanol was used as the negative control. Butylhydroxytoluene (BHT), butylhydroxyanisole (BHA), and ascorbic acid (vitamin C) were used as reference compounds. The test was repeated, and the following Formula (10) was used to calculate the percentage of inhibition (PI)

(10) $$\text{PI}\%=\frac{A_{0}-A}{A_{0}}\times 100$$

where PI is the percentage of inhibition, *A*_0_ is the optical density of the free radical (DPPH) solution in the absence of the extract (negative control), *A* is the absorbance of the free radical (DPPH) solution in the presence of the extract.

#### 2.10.2 Ferric Reducing Antioxidant Power (FRAP) Test

This method is based on the ability of an antioxidant to reduce ferric iron (Fe^3+^) in a potassium ferrocyanide (K_3_Fe(CN)_6_) complex to ferrous iron (Fe^2+^), as described by Oyaizu.^[^ ^48^ ^]^ The color goes from yellow (ferric) to blue‐green (ferrous) as a result of this change. The strength of this color change is measured at 700 nm using a spectrophotometer. To perform the assay, 10 µL of each sample at different concentrations is placed in three wells of a 96‐well plate. Next, 40 µL of phosphate buffer (pH 6.6) and 50 µL of a 1% potassium ferrocyanide solution (1 g K_3_Fe(CN)_6_ in 100 mL of water) are added to each well after being kept at 50 °C for 20 min; then, 50 µL of 10% trichloroacetic acid (TCA) and 40 µL of water are added. Then, 10 µL of 0.1% ferric chloride (FeCl_3_) is added. A spectrophotometer is then used to examine the plate and measure the amount of light it absorbs at 700 nm. Ascorbic acid was used as a standard, and the results were reported as the effective concentration at 50% (EC_50_), which is the level needed to lower the iron by 50%.

#### 2.10.3 Determination of Total Antioxidant Capacity (TAC)

This method measures the ability of the extracts to reduce molybdenum (Mo).^[^ ^49^ ^]^ The first step is to heat a water bath to 99 °C. You put 5 or 10 µL of extract (with a stock concentration of 25 mg mL^–1^) in a test tube and then 1 mL of ammonium molybdate (4 mM), 1 mL of sodium phosphate (28 mM), and 1 mL of sulfuric acid (0.6 M). The mixture is shaken, covered, and then put in an incubator at 95 °C for 90 min. The mixture is cooled to room temperature (20–30  min) after incubation. We measure the absorbance at 695 nm against a blank that contains only the reagents. We measured the total antioxidant capacity of the extracts in milligrams of ascorbic acid equivalents (mg EAA) per gram of dry extract. The results are based on a calibration curve (*y* = 0.0413x + 0.0209, *R* ^2^ = 0.9971). The following Formula (11) is used to figure out the total antioxidant capacity (TAC) as a percentage of inhibition Formula (11)

(11) $$\text{TAC}\left(\%\right)=\left[\frac{A_{\text{extract}}-A_{\text{control}}}{A_{\text{blank}}}\right]*100$$

where *A*_extract_ is the absorbance of the sample, *A*_control_ is the absorbance of the control (without extract), *A*_blank_ is the absorbance of the blank.

### 2.11 Study of the Antimicrobial Activity

The antimicrobial activity of the essential EO and extracts of *T. polium* was determined using the microdilution method in 96‐well microplates to identify the minimum inhibitory concentration (MIC).^[^ ^50^ ^]^ The MIC is the lowest concentration of the EO or extracts required to completely inhibit microbial growth during incubation, as indicated by the absence of visible growth. A series of dilutions was prepared from a stock solution of the EO or extracts dissolved in 10% DMSO, to obtain concentrations ranging from 5 to 0.93 × 10^2^ mg mL^–1^. These dilutions were prepared with a final volume of 100 µL for each concentration in Sabouraud broth for fungi and Mueller–Hinton medium for bacteria. Then, 100 µL of microbial inoculum, at a final concentration of 10^6^ CFU/mL for bacteria or 10^4^ CFU/mL for fungi, was added to each well. After 24 h of incubation at 37 °C, 10 µL of resazurin was added to each well to assess microbial growth. A color change from violet to pink, after a second 2‐h incubation at 37 °C, indicated the presence of microbial growth. The MIC was defined as the lowest concentration preventing this color change of resazurin. Growth and sterility controls were included in wells 11 and 12, respectively. The test was performed in duplicate for each extract and extract oil. The minimum bactericidal concentration (MBC) and minimum fungicidal concentration (MFC) were determined by taking 10 µL from each well that showed no visible growth and then spreading them on Mueller–Hinton agar for bacteria or Sabouraud broth for fungi, followed by incubation for 24  h at 37 °C. The lowest concentration resulting in a 99.99% reduction in CFU/mL compared to the control was considered the MBC or MFC.

### 2.12 Acute Toxicity

The acute toxicity study was conducted on albino mice (both males and females), weighing between 20 and 35 g. The animals were randomly divided into four groups (*n* = 6, with one male and one female per group) after a 14‐h fasting period. The control group received distilled water (10 mL kg^–1^), while groups 1, 2, and 3 were administered increasing doses of decoction (0.5, 1, and 2 g kg^–1^, respectively). After the initial weighing, a single dose of decoction was administered orally, and the mice were continuously observed for the first 10 h for any signs of acute toxicity, such as behavioral, physical changes, or signs of distress. Observations continued daily for 14 days, recording any clinical or behavioral changes indicative of toxicity. The monitored parameters included general appearance, behaviors (activity, restlessness, and lethargy), changes in excretory habits, and variations in body weight. In case of signs of severe toxicity, histopathological and biochemical examinations could be performed to determine the affected target organs. The study also aimed to determine the lethal dose (LD50) and the no‐observed‐adverse‐effect level (NOAEL) based on the observed symptoms and potential mortality.

### 2.13 Study of the Antidiabetic Activity In Vitro

The *α*‐amylase inhibitory activity of *T. polium* extracts was evaluated using a modified method based on the one described by Kamal et al.^[^ ^51^ ^]^ Increasing concentrations of *T. polium* extracts or acarbose (positive control) were prepared. Then, 250 µL of each sample was mixed with 250 µL of 0.02 M sodium phosphate buffer (pH 6.9) containing *α*‐amylase (240 U mL^–1^). The mixture was pre‐incubated at 37 °C for 20 min. After incubation, 250 µL of a 1% starch solution prepared in 0.02 M sodium phosphate buffer (pH 6.9) was added, and the reaction was allowed to proceed for 15 min at 37 °C. The enzymatic reaction was stopped by adding 1 mL of 2.5% dinitrosalicylic acid (DNS) reagent, and the tubes were heated in a water bath for 10 min. After cooling in an ice bath, the reaction mixture was diluted with 2 mL of distilled water, and absorbance was measured at 540 nm using a UV‐Vis spectrophotometer.

For the *α*‐glucosidase inhibition assay, the method described by Kee et al. was used with some modifications. A reaction mixture containing 150 µL of extract and 100 µL of 0.1 M phosphate buffer (pH 6.7) with *α*‐glucosidase (0.1 U mL^–1^) was incubated at 37 °C for 10 min. Then, 200 µL of 1 mM p‐nitrophenyl‐*α*‐D‐glucopyranoside (pNPG) substrate was added, and the reaction was continued at 37 °C for 30 min. The reaction was stopped by adding 1 mL of 0.1 M sodium carbonate (Na_2_CO_3_), and absorbance was recorded at 405 nm. Acarbose was used as a positive control.

To determine the type of inhibition, assays were performed with different concentrations of starch and pNPG in the presence of varying concentrations of the extract. The inhibition mechanism was analyzed using a Lineweaver‐Burk plot based on Michaelis‐Menten kinetics. The percentage of inhibition for both assays was calculated using the following Formula (12)

(12) $$\%\text{Inhibition}=\left[\frac{\left(Ac\;-Abc\;\right)-\left(As\;-Abc\;\right)}{\left(Ac\;-Abc\right)}\right]*100$$

where *As* represents the absorbance of the sample, *Ac* represents the absorbance of the control, *Abs* represents the absorbance of the blank sample, *Abc* represents the absorbance of the blank control.

### 2.14 Study of the Antidiabetic Activity In Vivo

#### 2.14.1 Animal Material

The antidiabetic activity in vivo was evaluated using Wistar rats (200–250 g) and albino mice (25–35 g), bred under ideal conditions (a 12‐h light/12‐h dark photoperiod at 22 ± 2 °C) with free access to water and food. The study complied with the guidelines of the Organisation for Economic Co‐operation and Development (OECD).^[^ ^52^ ^]^ Animals were acclimatized for 7 days prior to experimentation and assigned to groups using simple randomization. Each group had *n* = 6 animals in it. For rats, both males and females were employed (balanced), but only males were used for STZ induction.

#### 2.14.2 Streptozotocin‐Induced Diabetes

For diabetes induction, male albino mice (25–30 g, aged 6–8 weeks) were acclimatized for 1 week under controlled conditions (22 ± 2 °C, 12‐h light/dark cycle) with free access to standard rodent chow and water. Before streptozotocin (STZ) injection, the mice were fasted for 12–16 h but had free access to water. A fresh STZ solution was prepared by dissolving the compound in 0.1 M citrate buffer (pH 4.5) and administered via intraperitoneal injection at a dose of 40–60 mg kg^–^^1^ body weight (typically 50 mg kg^–1^).^[^ ^53^ ^]^ The injection was performed within 15 min of preparation to ensure compound stability. To prevent hypoglycemia, the mice were given a 5% dextrose solution in their drinking water for 24 h post‐injection.^[^ ^53^ ^]^

Blood glucose levels were monitored using a glucometer on the third and seventh days after STZ administration. Mice with fasting blood glucose (FBG) levels ≥250 mg dL^–^^1^ were considered diabetic and selected for further experimental evaluations. Physical signs such as weight loss, excessive thirst (polydipsia), and frequent urination (polyuria) were also observed to confirm diabetes. The diabetic mice were then maintained on a regular diet and used for pharmacological studies and metabolic/biochemical assessments; no histopathological examinations were performed in this study. This protocol provides a reliable and reproducible method for inducing diabetes in mice, making it suitable for further research.^[^ ^54^ ^,^ ^55^ ^]^

#### 2.14.3 Oral Glucose Tolerance Test (OGTT)

This assay is used to evaluate the antihyperglycemic effect of a test compound in vivo. Male Wistar rats are fasted for 12 h before the experiment to stabilize their baseline glucose levels. The animals are then divided into different groups: a control group receiving distilled water, a test group receiving the extract at a dose of 70 mg kg^–1^, and a standard group receiving glibenclamide at a dose of 2 mg kg^–1^. At baseline (*t*_0_), blood glucose is measured using a glucometer before administering the test compound or control. Thirty minutes later, a glucose load (2 g kg^–^^1^) is administered orally, and blood glucose levels are measured at 30, 60, 90, and 120 min after glucose administration. A reduction in postprandial glucose levels compared to the control group indicates the potential antihyperglycemic activity of the tested extract.^[^ ^56^ ^,^ ^57^ ^]^

#### 2.14.4 Biochemical Parameters

On day 30, blood samples were collected by puncture of the retro‐orbital plexus using capillary tubes containing EDTA. The samples were centrifuged at 3000 rpm for 10 min at 4 °C, and the plasma was collected for analysis. The following biochemical parameters were measured using commercial assay kits: creatinine, serum glucose, aspartate aminotransferase (AST), triglycerides, uric acid, total protein, urea, cholesterol, and alanine aminotransferase (ALT). Glucose was quantified using the glucose oxidase assay kit (BioVision). AST and ALT were measured using enzymology kits for aspartate aminotransferase (AST) and alanine aminotransferase (ALT) (Abcam), respectively. Triglycerides were determined with the triglyceride assay kit (Sigma–Aldrich), and cholesterol was measured with the cholesterol assay kit (Abcam). Creatinine and urea levels were measured using creatinine and urea assay kits (Cayman Chemical), uric acid with the uric acid assay kit (Sigma–Aldrich), and total protein with the total protein assay kit (Thermo Fisher). All analyses were performed according to standard laboratory procedures.

### 2.15 Statistical Analysis

Analyses were performed using GraphPad Prism 9 (version 9.5.1, San Diego, CA, United States). Results are expressed as mean ± SEM, with n specified in the figure/table legends. Normality and homogeneity of variances were assessed (Shapiro–Wilk; Levene/Brown–Forsythe). Group comparisons used one‐way ANOVA followed by Tukey's post hoc test; for repeated measures, repeated‐measures ANOVA with Tukey post hoc was applied. Statistical significance was set at *p* < 0.05. In vitro assays were conducted with at least technical triplicates and independently repeated; for in vivo experiments, n per group is indicated.

## 3 Results and Discussion

### 3.1 Quality Control of Plant Material

The phytochemical analysis of *T. polium* plant material focused on several key parameters used to evaluate the stability, safety, and efficacy of the final product. Such parameters are moisture content (MC), pH, titratable acidity, and total ash content. These are the primary criteria that must be met to ensure the product meets quality standards and can be used as a medicine or food. **Table** **2** presents the results obtained for these parameters.

**Table 2:** Quality control of *T. polium* plant material.

MC [%]	pH	Acidity	Ash
7.12 ± 0.09	7.2 ± 0.02	0.31 ± 0.02	11.75 ± 0.01

*The T. polium sample has a moisture content of 7.12%*. Such a low value indicates that the plant material was dried appropriately, which is necessary to prevent the risk of biological degradation during storage. High moisture levels may stimulate the growth of microbes and fungi, which can trigger changes in therapeutic effects and product contamination. As a general rule, it is recommended to maintain the moisture level of dried plants at a level that does not exceed 10% to minimize these risks without affecting the active compounds.^[^ ^58^ ^]^ A low moisture content, as recorded in this study, is thus an assurance of product stability, particularly because this parameter is a significant requirement for the long‐term stability of bioactive compounds found in plants.

The pH of the sample was 7.2, which showed that the plant material was neutral. A neutral pH can be regarded as an indicator of the harmony in the composition of a plant's chemical components. This pH is also desirable because it can be used in medicinal preparations, where it may be beneficial to reduce the likelihood of irritation, as well as because it is compatible with many extraction techniques that can be sensitive to changes in pH. In addition, a neutral pH is stable, indicating that the plant would be highly adaptable to pharmaceutical formulation methods, where pH extremes may result in a changed therapeutic effect or instability of the extracts.^[^ ^59^ ^]^

The titratable acidity result was 0.31%, indicating a low content of organic acids in the sample. Such acidity is especially beneficial for the medicinal and food use of *T. polium*, as it helps to make the plant less astringent and bitter, thereby enhancing its palatability as a food or medicine product. Low titratable acidity may also reflect low levels of undesirable compounds that may impact the efficacy or palatability of the product.^[^ ^60^ ^]^

Lastly, a total ash content was determined to be 11.75%. This index indicates that the sample contains moderate amounts of minerals, which are common in medicinal plants that are often used as sources of critical minor elements for the body. The amount of ash, or the nonorganic mineral fraction that remains after burning, is an indication of the abundance of elements like calcium, magnesium, and potassium, all of which are vital constituents of many biological processes. Nevertheless, a very high ash content may indicate contamination or undesirable enrichment with cultivation medium or minerals from the drying process. A moderate level, such as in the present case, is desirable, as it guarantees, on the one hand, the nutritional richness of the product and, on the other, the absence of undue contamination.^[^ ^61^ ^]^

### 3.2 Determination of Mineral Composition by ICP‐AES

*T. polium* was analyzed regarding its mineral content (trace elements (Fe and Cu) and heavy metals (As, Cr)). **Table** **3** shows the obtained results. The heavy metal content analysis in *T. polium* indicated that this plant is not predominantly polluted, as the content of crucial metals, namely arsenic (As), copper (Cu), iron (Fe), and chromium (Cr), is much lower than the safety levels. The concentration of arsenic is also very low in quantity, at 0.0058 mg g^–^^1^, which is well below the toxicity levels set for plants. Farooq et al.^[^ ^62^ ^]^ stated that the maximum level of arsenic in medicinal plants should not exceed 0.1 mg g^–^^1^ to be considered toxic, and the level in this study is significantly lower, indicating that *T. polium* is safe regarding arsenic contamination. Arsenic refers to a poisonous metalloid, and long‐lasting exposure to significant levels might cause severe health complications, including carcinogenic harm. The trace found in this plant, however, is so negligible that it indicates a very low risk to human health. Similarly, copper (Cu), at 0.0030 mg g^–^^1^, is well below the danger level. Copper is a micronutrient essential for plant growth, but excessive amounts can also be toxic to plants. According to Cruz et al.^[^ ^63^ ^]^ a copper level above 20 mg kg^–^^1^ may be harmful to plants and human health; however, in *T. polium*, it is much lower and therefore not harmful. This low copper concentration is likely an indication of the plant's natural ability to absorb nutrients, and there is no reason to suspect contamination or poisoning.

**Table 3:** Trace elements and heavy metals of *T.polium*.

Metals	Mineral composition content [mg g^–^^1^]
Arsenic (As)	0.0058
Iron (Fe)	0.2706
Copper (Cu)	0.0030
Chromium (Cr)	0.0008

The content of iron (0.2706 mg g^–^^1^) corresponds to the usual content in medicinal plants. Iron is a vital nutrient for both plants and humans, and it plays a significant role in biology, where it is utilized in photosynthesis and chlorophyll synthesis. As stated by Lapaz et al.^[^ ^64^ ^]^ moderate amounts of iron in plants are normal and do not pose any health hazards, unless the concentrations become excessively high. The concentration of iron identified in this research is far below the safety threshold and likely represents the optimal level required by the plant to grow and metabolize. Lastly, the concentration of chromium (Cr) is negligible (0.0008 mg g^–^^1^) and poses no threat to either plants or humans. Hexavalent chromium (Cr^6+^) is highly toxic, but its trivalent chromium (Cr^3+^) form, which is predominant in natural environments, is relatively less harmful. Researchers, such as Qureshi et al.^[^ ^65^ ^]^ have demonstrated that plants at low concentrations primarily take up chromium, and at these concentrations, there is no threat of chromium toxicity.

In general, the heavy metal profile of *T. polium* indicates that the plant is growing in a relatively unpolluted area. The levels of these metals are far lower than the toxic levels set by regulatory agencies, such as the World Health Organization (WHO) and European Union standards; thus, it is a safe plant to be used medicinally and therapeutically. These findings align with those of past research, including Shirazi‐Tehrani et al.^[^ ^66^ ^]^ who argue that the safety of medicinal plants depends on their low heavy metal content. Such low concentrations indicate that *T. polium* not only lacks substantial pollution but also has a good proportion of the most essential nutrients, which qualifies it as a candidate herbal medicine without the risk of heavy metal poisoning.

### 3.3 Phytochemical Study

The initial chemical screening of *T. polium* yields valid data regarding the pattern of bioactive compounds in the plant (**Table** **4**), but, as these results are based on qualitative chemical reactions, they should be interpreted as indicative rather than definitive. The outcomes reveal a wide range of secondary metabolites, all of which contribute to the plant's pharmacological effects, specifically its antioxidant, anti‐inflammatory, antimicrobial, and analgesic properties. The presence of polysaccharides, as noted in earlier research,^[^ ^67^ ^]^ underscores the immune‐modulatory potential of the plant and its antioxidant activity. Polysaccharides, particularly those with immunostimulatory and prebiotic properties, play a role in modulating immune responses and gut health, suggesting that *T. polium* may have therapeutic applications in treating diseases related to oxidative stress and immune dysfunction.^[^ ^68^ ^]^

**Table 4:** Phytochemical screening of the aerial parts of *T. polium*.

Chemical groups			Observations
**Primary metabolites**	Polysaccharides		+++
	Lipids		++
	Proteins	Biuret reaction	+
		Xanthoproteic reaction	–
	Reducing sugars		+
**Secondary metabolites**	Tannins	Gallic	+
		Catechic	++
	Flavonoids	Flavones	++
		Leucoanthocyanins	++:
	Anthocyanins		+
	Saponins		++
	Alkaloids	Mayer	+++
		Dragendorff	+++
		Wagner	+++
	Reducing components		+
	Oses and holosides		++
	Mucilages		++
	Sterols and triterpenes		+

+++ very abundant; ++ : abundant; + low; − absent.

The identification of lipids is also consistent with previous studies,^[^ ^10^ ^]^ indicating that lipids play a role in cellular protection by maintaining membrane integrity and providing antioxidant effects. Although present in moderate amounts, lipids contribute to the plant's therapeutic potential by protecting against oxidative damage and supporting cellular repair mechanisms. The detection of proteins in low quantities aligns with the findings of previous studies,^[^ ^69^ ^]^ which identified proteins but did not consider them to be dominant components of the plant's chemical profile. The adverse xanthoproteic reaction further suggests the absence of aromatic amino acids such as tyrosine, which are often precursors of nitrogenous bioactive compounds.

The presence of tannins, especially catechic tannins and gallic acid, is one of the most striking results of the present study and supports earlier claims^[^ ^59^ ^]^ about the involvement of tannins in antioxidant and antimicrobial processes. Tannins are polyphenolic compounds that have been attributed to scavenging free radicals and exhibiting antimicrobial effects, thereby supporting the traditional use of *T. polium* in the treatment of infections and diseases related to oxidative stress. Likewise, flavonoids, flavones, and leucoanthocyanins were also found in considerable amounts, which contribute to the plant's antioxidant activity. Flavonoids have anti‐inflammatory activity and can neutralize free radicals, which further supports the therapeutic potential of *T. polium* in inflammatory diseases.^[^ ^70^ ^]^ Their anthocyanin content, albeit lower, suggests that these compounds may also contribute to the plant's antioxidant effect, particularly in supporting vascular health.

The presence of saponins was also identified, which is essential because they are known to possess antimicrobial and anti‐inflammatory properties, as found by Sharifi‐Rad et al.^[^ ^58^ ^]^ Saponins have membrane‐stabilizing effects, which can be utilized to target microbial cells, thereby enhancing the antimicrobial properties of the plant. Besides these properties, saponins have been discussed as modulating immune responses and lowering lipid levels, thereby confirming the potential of *T. polium* in treating metabolic disorders and inflammation.

Mayer, Dragendorff, and Wagner reagents confirmed the high levels of alkaloids, which align with the currently known pharmacological properties of the plant, including analgesic, antimicrobial, and anti‐inflammatory activities.^[^ ^71^ ^]^ Alkaloids play a crucial role in pain relief and antimicrobial activity, making them a vital compound to consider for the therapeutic usefulness of *T. polium* in treating pain and controlling infections. The observed reducing compounds indicate the antioxidant potential of the plant by donating electrons, which aligns with its purpose to neutralize free radicals. Additionally, the presence of oses and holosides, as well as mucilages, strengthens the potential prebiotic action of *T. polium*, which may promote gut health and immune modulation, as suggested by the data of Ardestani et al.^[^ ^72^ ^]^

Lastly, sterols and triterpenes, although present in smaller quantities, contribute to the plant's anti‐inflammatory and lipid‐modulating effects. These substances, which have been linked to lowered cholesterol levels and protection against chronic inflammation, substantiate the therapeutic usefulness of *T. polium* in the treatment of metabolic and inflammatory diseases, as observed in other experiments.^[^ ^73^ ^]^

### 3.4 Extraction and Yield of EO

Several studies have been conducted on the EO of *T. polium* to evaluate its chemical composition and biological properties. The EO yield of *T. polium* was estimated at 1.65% (v/w). This result is similar to that reported in a study conducted in Algeria, where the essential oil of *T. polium* from Mount Tessala was extracted using hydrodistillation, yielding an average of 1.66% ± 0.12.^[^ ^74^ ^]^ In Morocco, another study focused on the phytochemical composition and biological activities of the essential oil of *T. polium* subsp. polium, obtaining a yield of 0.18% ± 0.02.^[^ ^75^ ^]^

The EO of *T. polium* exhibits distinct organoleptic characteristics, particularly in terms of color and odor (**Table** **5**). For instance, the EO of *T. polium* is characterized by a yellowish color and a very strong and persistent odor.^[^ ^76^ ^]^ The density of the essential oil was found to be 0.860 g mL^–^^1^. It is generally observed that these EO have a density lower than that of water, which causes them to float when separated by hydrodistillation.

**Table 5:** Yield, organoleptic, and physicochemical characteristics of the essential oil of *T. polium*.

Yield [%]	Density [g mL^–^^1^]	Color	Odor
1.65 ± 0.10	0.860 ± 0.009	Yellowish	Very strong and persistent

These variations in yields and organoleptic properties can be attributed to various factors, including the plant subspecies, growth conditions, drying methods, and extraction techniques used.

### 3.5 Analysis and Identification of Essential Oils by GC–MS

**Figure 1:**
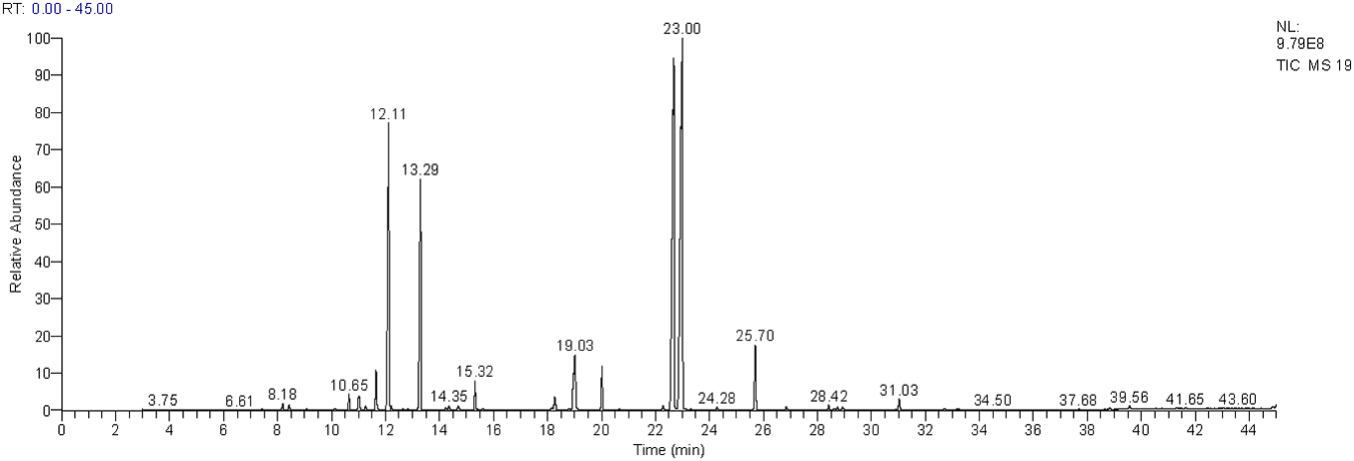
Gas chromatography‐mass spectrometry (GC/MS) profile of EO extracted from the tops of *T. polium* L.

The analysis of EO extracted from the leaves of *T. polium*. using GC–MS, as presented in **Figure** **1**, reveals a particularly rich and diverse chemical composition.

In total, 32 chemical compounds were identified, representing ≈99.62% of the total composition of the essential oils. Among them, thirteen compounds are present at concentrations above 1% (**Table** **6**). The major compounds are carvacrol (28.10%), thymol (26.28%), and *γ*‐terpinene (12.11%). These compounds are well‐known for their antimicrobial and antioxidant properties.^[^ ^77^ ^]^

**Table 6:** Chemical composition of the essential oil of *T. polium*.

N°	R.T.	KI (experimental)	KI (Adams)	Compound	Mass	Formula	Area [%]
1	7.42	890	895	3‐Heptanone	114	C_7_H_14_O	0.06
2	8.18	924	930	*α*‐Thujene	136	C_10_ H_16_	0.25
3	8.43	939	939	*α*‐Pinene	136	C_10_ H_16_	0.18
4	10.13	979	979	*β*‐Pinene	136	C_10_ H_16_	0.06
5	10.65	991	990	Myrcene	136	C_10_ H_16_	0.64
6	11.01	988	983	3‐Octanone	136	C_10_ H_16_	0.87
7	11.26	1005	1002	*α*‐Phellandrene	136	C_10_ H_16_	0.17
8	11.65	1017	1017	*α*‐Terpinene	136	C_10_ H_16_	1.71
9	12.11	1027	1026	*o*‐Cymene	134	C_10_ H_14_	15.59
10	12.22	1032	1029	*β*‐Phellandrene	136	C_10_ H_16_	0.10
11	12.63	1033	1031	1,8‐Cineole	154	C_10_ H_18_O	0.05
12	13.29	1059	1059	*γ*‐Terpinene	136	C_10_ H_16_	12.11
13	14.24	1088	1088	Terpinolene	136	C_10_ H_16_	0.10
14	14.35	1073	1070	cis‐Sabinene hydrate	154	C_10_ H_18_O	0.20
15	14.69	1090	1091	*ρ*‐Cymenene	132	C_10_ H_12_	0.19
16	15.32	1097	1096	Linalool	154	C_10_ H_18_O	1.31
17	15.61	1120	1121	cis‐*ρ*‐Menth‐2‐en‐1‐ol	154	C_10_ H_18_O	0.06
18	18.16	1165	1169	Borneol	154	C_10_ H_18_O	0.14
19	18.27	1177	1177	Terpinen‐4‐ol	154	C_10_ H_18_O	0.74
20	18.80	1180	1179	m‐Cymen‐8‐ol	150	C_10_ H_14_O	0.07
21	19.03	1189	1188	*α*‐Terpineol	154	C_10_ H_18_O	4.38
22	20.01	1243	1244	Carvacrol, methyl ether	164	C_11_ H_16_O	1.93
23	22.68	1290	1290	Thymol	150	C_10_ H_14_O	26.28
24	23.00	1299	1299	Carvacrol	150	C_10_ H_14_O	28.10
25	24.28	1356	1359	Eugenol	164	C_10_ H_12_O_2_	0.13
26	25.70	1418	1419	(E)‐Caryophyllene	204	C_15_H_24_	3.19
27	26.85	1454	1454	*α*‐Humulene	204	C_15_ H_24_	0.16
28	28.42	1500	1505	*β*‐Bisabolene	204	C_15_ H_24_	0.20
29	28.75	1524	1523	*δ*‐Cadinene	204	C_15_ H_24_	0.10
30	28.93	1526	1522	*β*‐Sesquiphellandrene	204	C_15_ H_24_	0.49
31	31.03	1582	1583	Caryophyllene oxide	220	C_15_ H_24O_	0.63
32	44.88	2430	2436	6‐Deoxy‐Taxodione	300	C_20_ H_28_O_2_	0.23
				Identified compounds [%]			99.62
				Monoterpenes [%]			30.97
				Oxygenated monoterpenes [%]			63.12
				Sesquiterpenes [%]			4.12
				Oxygenated sesquiterpenes [%]			0.63
				Diterpenes [%]			0.23

O‐Cymene (15.59%) is a precursor to thymol and carvacrol, playing a key role in the aroma and biological activity of the essential oils. Linalool (1.31%), terpinen‐4‐ol (0.74%), and terpineol (4.38%) are oxygenated monoterpenes, often associated with antifungal and relaxing properties.^[^ ^78^ ^,^ ^79^ ^]^

The main dominant chemical classes identified in the sample are hydrocarbon monoterpenes (30.97%), including notably *γ*‐terpinene, *α*‐terpinene, *α*‐pinene, *β*‐pinene, and o‐cymene. Oxygenated monoterpenes represent 6.92% of the composition and include compounds such as linalool, terpinen‐4‐ol, terpineol, and borneol. Monoterpenic phenols constitute the majority fraction, accounting for 56.20%, primarily represented by thymol and carvacrol. Sesquiterpenes are present at 4.12%, including (E)‐caryophyllene, while oxygenated sesquiterpenes constitute 0.63%, including caryophyllene oxide. Finally, diterpenes, represented by taxodione, are present in small quantities (0.23%).

These findings support earlier research demonstrating that the essential oil of *T. polium* is abundant in phenolic compounds, recognized for their strong antimicrobial and antioxidant characteristics.^[^ ^80^ ^]^ Hydrocarbon monoterpenes comprise 30.97% of the essential oil, with notable levels of *γ*‐terpinene (12.11%), o‐cymene (15.59%), *α*‐terpinene (1.71%), and *α*‐pinene (0.18%). These compounds contribute to the characteristic odor and biological activity of the EO.^[^ ^81^ ^]^ Oxygenated monoterpenes make up 63,12% of the composition, with carvacrol (28.10%), thymol (26.28%), *α*‐terpineol (4.38%), and terpinen‐4‐ol (0.74%) as notable compounds. These molecules are often associated with antibacterial and antifungal properties.^[^ ^82^ ^]^ Hydrocarbon sesquiterpenes and oxygenated sesquiterpenes represent 4.12% and 0.63%, respectively, including caryophyllene (3.19%) and caryophyllene oxide (0.63%), which are known for their anti‐inflammatory effects and their role in the stability of the essential oil.^[^ ^83^ ^]^ Finally, the presence of taxodione (0.23%), a diterpene known for its cytotoxic properties, is a noteworthy element of this analysis.^[^ ^84^ ^]^

### 3.6 Distribution of the Main Chemical Families of T. polium EO

Table 6 also illustrates the distribution of the prominent chemical families in *T. polium* essential oil. The oil's biological properties can be attributed to the fact that oxygenated monoterpenes comprise the majority of its composition, followed by hydrocarbon monoterpenes.

Phenolic compounds make up most of the essential oil of *T. polium*, with carvacrol (28.10%) and thymol (26.28%) being the most common. These molecules are highly effective in combating free radicals, which helps reduce oxidative stress, a major contributor to chronic diseases such as diabetes and heart disease.^[^ ^85^ ^]^ Their combined effects with other known monoterpenes, such as o‐cymene (15.59%) and *γ*‐terpinene (12.11%), make this activity stronger because they are biosynthetic precursors of thymol and carvacrol.^[^ ^86^ ^]^ There is considerable evidence that *T. polium* can help manage diabetes, and this appears to be due to its high content of phenolic and sesquiterpene compounds. It has been suggested that carvacrol and thymol may enhance insulin function, lower blood sugar levels, and inhibit digestive enzymes from breaking down carbohydrates.^[^ ^12^ ^]^ Additionally, sesquiterpenes such as caryophyllene (3.19%) and caryophyllene oxide (0.63%) protect the pancreas by reducing systemic inflammation, a major contributor to the development of type 2 diabetes.^[^ ^10^ ^,^ ^87^ ^]^ *T. polium* has compounds that could be useful in treating metabolic diseases, as chronic inflammation exacerbates them. Carvacrol and thymol are known to inhibit the production of pro‐inflammatory cytokines, such as TNF‐*α* and IL‐6, thereby reducing the inflammatory response.^[^ ^88^ ^]^ Additionally, *β*‐caryophyllene and caryophyllene oxide contribute to this effect by modulating key inflammatory pathways, particularly NF‐*κ*B and MAPK. This lowers the activation of macrophages and the production of inflammatory mediators.^[^ ^89^ ^,^ ^90^ ^]^ *T. polium* essential oil contains a high concentration of carvacrol and thymol, making it highly effective against various harmful bacteria.^[^ ^91^ ^]^ These chemicals break down the membranes of microorganisms, allowing essential cell components to leak out and preventing them from functioning or killing them. Additionally, the presence of taxodione (0.23%) suggests potential cytotoxic activity, as studies have demonstrated that this type of diterpene can induce cell death in specific cancer cell lines.^[^ ^92^ ^]^ The chemical composition of *T. polium* EOs varies depending on their origin and prevailing weather conditions. In Iran, for instance, *α*‐cadinol (46.2%) and caryophyllene oxide (25.9%) are the most common essential oils. These oils have more anti‐inflammatory and neuroprotective effects. In Tunisia, on the other hand, samples high in sabinene and linalool have an antibacterial and relaxing impact.^[^ ^93^ ^]^ This chemical variability underscores the importance of thoroughly describing each essential oil before using it for any therapeutic purpose.

### 3.7 Extraction and Characterization of Polyphenolic Compounds from T. polium

#### 3.7.1 Extraction Yield

**Table** **7** presents the results of two different extraction methods; decoction, aqueous Soxhlet, and ethanolic Soxhlet, which were used to determine the amount of phenolic compounds that could be extracted from *T. polium*.

**Table 7:** Yields of T. polium extracts.

Plant	Decoction	Aqueous Soxhlet	Ethanolic Soxhlet
Mass of plant material [g]	30	30	30
Mass of extract [g]	7.20	4.90	5.33
Extraction yield [%]	24.00	16.33	17.76

The extraction yield by decoction was 24%. This method favors the extraction of water‐soluble compounds, notably flavonoids and phenolic acids, as well as other polar constituents. This method is popular because it is simple and effective in extracting active plant ingredients, especially those that combat inflammation and protect cells from damage.^[^ ^94^ ^]^ The aqueous Soxhlet extraction, on the other hand, yielded 16.33%, which is slightly lower but still competitive. This suggests that this method also allows for a good concentration of bioactive compounds, especially those that are water‐soluble.^[^ ^8^ ^]^ The hydroethanolic Soxhlet method yielded 17.76%, which is also a good result. Using a hydroethanolic solvent enables the extraction of both hydrophilic and lipophilic compounds from a plant. This makes the method more versatile for complex plants, such as *T. polium*.^[^ ^95^ ^]^

The study's results align with those of earlier studies, which have demonstrated that the method used to extract something can significantly impact the amount produced. For instance, Al Salloum et al.^[^ ^96^ ^]^ conducted a study that demonstrated that Soxhlet extraction with mixed solvents (water and ethanol) yields a better result for certain types of metabolites than methods that use only water or a single solvent. Similarly, a study by Abdullah et al.^[^ ^14^ ^]^ found that decoction was one of the most effective methods for extracting flavonoids from various medicinal plants. This backs up the findings of this study. While decoction offers the highest extraction from *T. polium*, both aqueous and ethanolic Soxhlet techniques also yield notable results, each presenting distinct advantages based on extraction goals and targeted components.

#### 3.7.2 Determination of Total Polyphenolic Compounds

**Figure 2:**
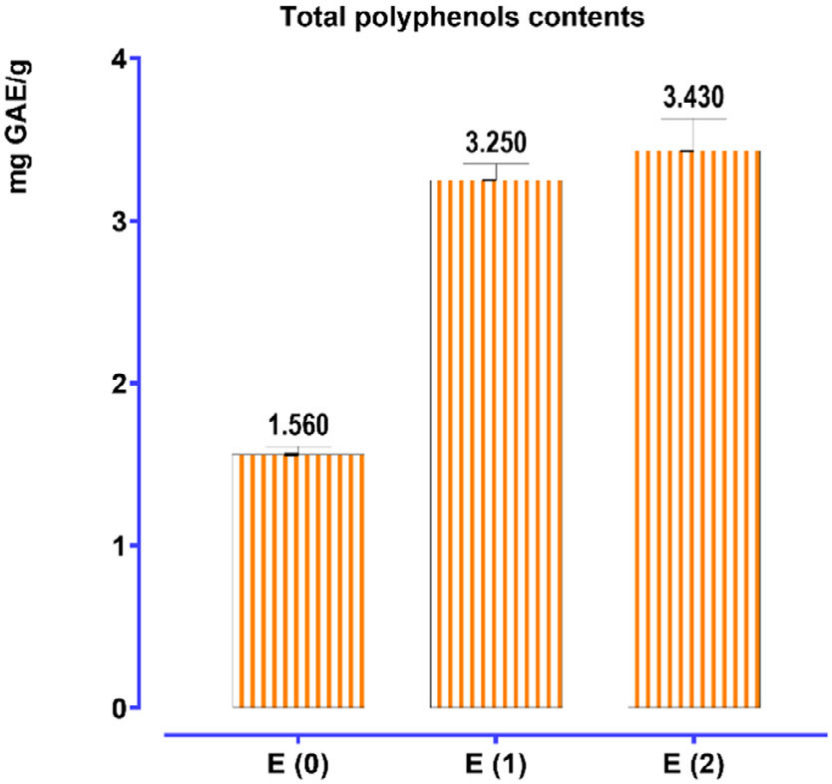
Determination of total polyphenolic compounds in *T. polium*.

The assessment of total polyphenol content in *T. polium* extracts was conducted utilizing the Folin‐Ciocalteu method, with a calibration curve established from varying amounts of gallic acid. The derived equation, *Y* = 0.077X + 0.001, with a coefficient of determination (*R* ^2^) of 0.996, demonstrates a strong correlation between polyphenol concentrations and absorbances recorded at 760 nm, thus confirming the precision of the observations. This colourimetric technique is extensively employed for assessing total polyphenols in plant extracts owing to its sensitivity and reproducibility. The data analysis indicates that polyphenol concentration fluctuates based on the extraction method used (**Figure** **2**). The ethanolic extract exhibits the maximum concentration at 3.430 ± 1.65 mg GAE/g, followed by the aqueous Soxhlet extract at 3.250 ± 0.25 mg GAE/g; however, the decoction extract displays a lower concentration of 1.560 mg GAE/g. The discrepancies can be elucidated by the solubility of polyphenols in various solvents and the thermal sensitivity of specific bioactive components. Ethanol, acting as a cosolvent with water, enhances the extraction of polyphenols by interacting with both hydrophilic and moderately lipophilic molecules, thereby facilitating their solubilization and diffusion from the plant matrix. Numerous investigations have shown that alcoholic extracts of *T. polium* exhibit superior extraction of flavonoids and phenolic acids compared to aqueous extracts alone.^[^ ^20^ ^]^

The aqueous extract obtained by Soxhlet also exhibits a relatively high polyphenol content, which can be attributed to the efficiency of this extraction method, as it uses constant solvent renewal to maximize the extraction of bioactive compounds. However, the extract obtained by decoction shows a lower polyphenol content, which may be due to the thermal degradation of certain phenolic compounds that are sensitive to heat. This result is consistent with the findings of Bahramikia et al.^[^ ^10^ ^]^ which demonstrated that the high temperatures used in decoction can lead to hydrolysis or oxidation of polyphenols, thereby reducing their concentration in the final extract. Nonetheless, decoction remains a commonly used extraction method in traditional medicine, particularly in Morocco, due to its simplicity and accessibility.

Previous studies on *T. polium* have similarly reported that alcoholic extraction allows for the extraction of a greater quantity of polyphenols due to the increased solubility of these compounds in ethanol.^[^ ^70^ ^]^ Indeed, water alone is sufficient for extracting hydrophilic compounds, such as flavonoids and certain tannins; however, the addition of ethanol enhances the extraction of less polar polyphenols, notably phenolic acids and flavones.^[^ ^97^ ^]^ This observation was confirmed by Al‐Naemi et al.^[^ ^20^ ^]^ who emphasized that ethanolic extraction maximizes the release of bioactive compounds from *T. polium*, thereby enhancing its antioxidant and anti‐inflammatory potential.

The lower concentration observed in the decoction extract (1.560 mg GAE/g) could be attributed to the thermal degradation of certain heat‐sensitive polyphenols, such as anthocyanins and certain flavonoids.^[^ ^58^ ^]^ Several studies on Moroccan medicinal plants have highlighted the decrease in polyphenol content after prolonged exposure to high temperatures, which could explain the differences between the extraction methods.^[^ ^31^ ^]^

#### 3.7.3 Determination of Flavonoids

**Figure 3:**
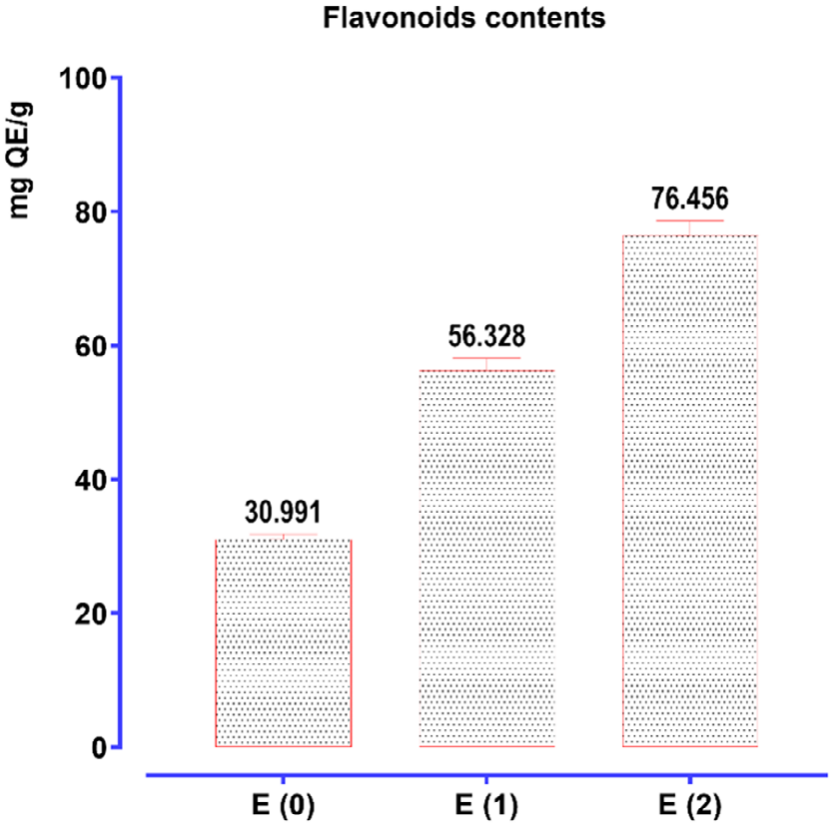
Determination of flavonoids in *T. polium*.

The quantification of flavonoids in *T. polium* was performed using quercetin as the reference standard, with a calibration curve defined by the equation *y* = 0.0421*x* − 0.0763 (*R* ^2^ = 0.9512). The results, as shown in **Figure** **3**, reveal particularly high concentrations of flavonoids, with a clear distinction among the three extraction methods. The hydro‐alcoholic extract exhibits the highest flavonoid content (76.456 mg QE/g DW), followed by the aqueous extract (56.328 mg QE/g DW), and finally the decoction extract (30.991 mg QE/g DW).

The influence of ethanol can be ascribed to the alcoholic extract with the highest flavonoid concentration. Ethanol is a polar solvent that efficiently extracts both lipophilic and hydrophilic polyphenolic flavonoids, hence enhancing the yield of bioactive chemicals. Numerous studies have demonstrated that alcoholic solvents are particularly effective in extracting flavonoids from many medicinal plants. A survey by Javadpour et al.^[^ ^98^ ^]^ showed that ethanol facilitates flavonoid extraction in *T. polium*, and the incorporation of water augments the solubility of glycosylated flavonoids. This conclusion aligns with observations made by other writers about the efficacy of alcoholic extracts in isolating flavonoids.

The aqueous extract exhibits a comparatively elevated content of flavonoids, aligning with findings from prior investigations. Water‐soluble flavonoids, including glycosylated flavonoids, can be efficiently extracted using water as the solvent. Sharififar et al.^[^ ^99^ ^]^ noted that aqueous extraction facilitated the retrieval of flavonoids from plants such as *T. polium*, which had considerable antioxidant and anti‐inflammatory activities. Utilizing water as a solvent is thus beneficial for extracting flavonoids that are not entirely soluble in pure organic solvents.

Conversely, the decoction extract demonstrates a markedly reduced content of flavonoids. This may be ascribed to the heat breakdown of flavonoids during boiling. Heat‐sensitive flavonoids may decompose at elevated temperatures, resulting in less bioactivity and a decrease in their overall content in the extract. Sharififar et al.^[^ ^99^ ^]^ demonstrated that applying heat during the decoction process can degrade flavonoids in several medicinal plants, including *T. polium*. This phenomenon arises from the heat sensitivity of some flavonoids, which restricts their extraction during the decoction process. Therefore, this extraction technique is inferior to cold or moderate‐temperature techniques for flavonoid recovery.

The flavonoid concentrations observed in this study (up to 76.456 mg QE/g DW in the alcoholic extract) are significantly higher than those reported in prior scientific investigations. A survey of *T. polium* in Turkey revealed flavonoid contents ranging from 40 to 50 mg g^–^^1^ in alcoholic extracts.^[^ ^100^ ^]^ This disparity may be ascribed to fluctuations in environmental conditions, harvesting times, and extraction techniques, all of which affect the flavonoid content in medicinal plants.

#### 3.7.4 Determination of Condensed Tannins

**Figure 4:**
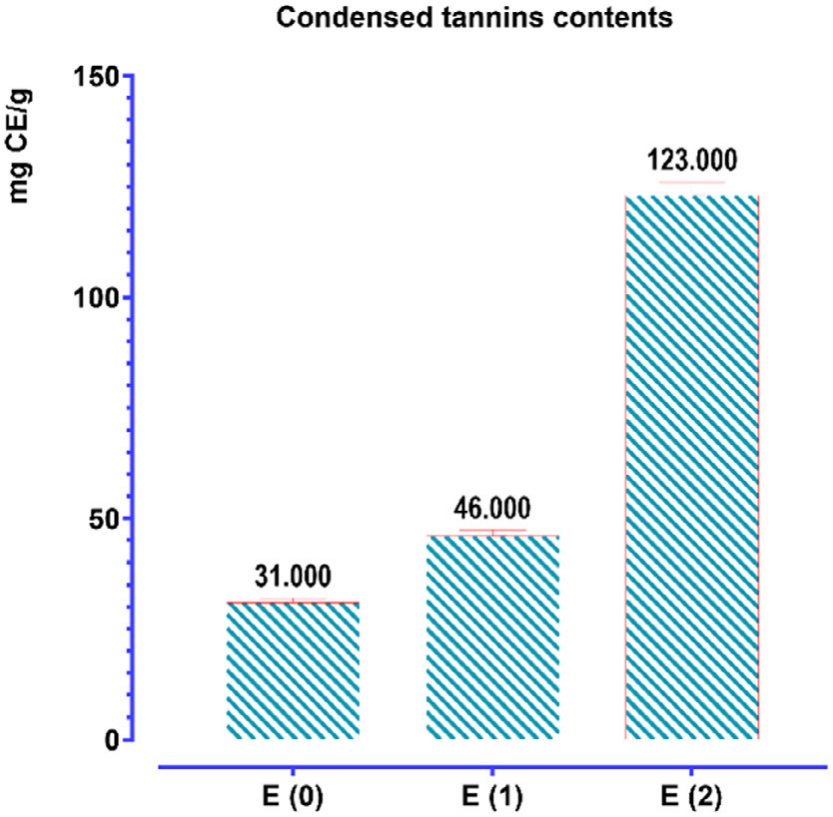
Quantification of total tannins in *T. polium*.

The results (**Figure** **4**) for the concentration of condensed tannins in *T. polium* extracts indicate substantial quantities of tannins, with significant variability based on the extraction method employed. The measurement of condensed tannins was conducted utilizing the catechin standard method, with a concentration range of 0–300 μg mL^–^^1^, enabling the expression of tannin concentrations in micrograms of catechin equivalent per milligram of extract (μg CE/mg extract). The calibration curve follows the equation *y* = 0.0027x—0.1232, exhibiting a high determination coefficient of *R* ^2^ = 0.9872, which indicates excellent linearity and reliability of the method.

The results show that the ethanolic extract of *T. polium* contains the highest amount of condensed tannins, with a concentration of 123 mg CE/g of dry matter (DM), indicating an efficient extraction of these compounds by ethanol, a solvent well‐known for its ability to dissolve polyphenols such as tannins. This high concentration observed in our ethanol extract is corroborated by our quantitative data and is consistent with previous studies reporting high polyphenol content in *T. polium*.^[^ ^58^ ^]^

On the other hand, the aqueous extract contains a more moderate amount of tannins, 46 mg CE/g DM. Although water is generally considered a good solvent for extracting a wide variety of polyphenolic compounds, its efficiency in extracting tannins is usually lower compared to alcoholic solvents, as shown by research on other medicinal plants.^[^ ^101^ ^]^ This could be due to the relatively lower solubility of tannins in water compared to ethanol.

Finally, the decoction extract of *T. polium* exhibits the lowest concentration of condensed tannins, with 31 mg CE/g DM. This low concentration could be attributed to the partial thermal degradation of tannins during the decoction process, a phenomenon often observed during high‐temperature extraction.^[^ ^102^ ^]^ Tannins are heat‐sensitive, which can lead to a reduced yield of bioactive compounds.

#### 3.7.5 Analysis and Identification of Compounds in T. polium Extracts by HPLC/UV‐ESI‐MS

**Figure 6:**
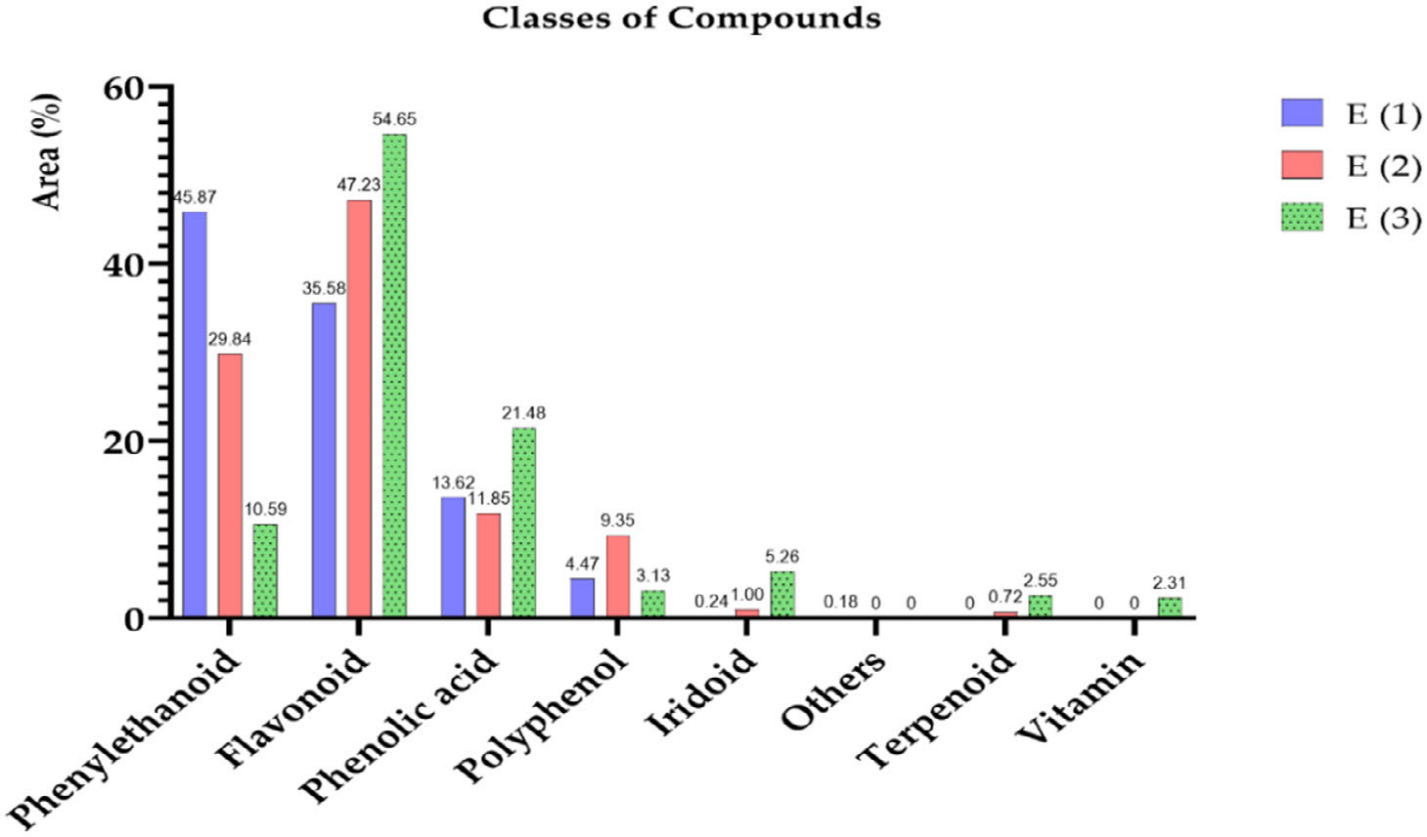
Distribution of different classes of compounds in the analyzed samples.

The chromatographic study combining HPLC/UV and ESI‐MS, illustrated in **Figure** **5**, enabled a comprehensive mapping of phenolic and nonphenolic compounds present in the different extracts of *T. polium*. The three extraction methods—aqueous decoction (E0), aqueous Soxhlet extraction (E1), and hydroethanolic Soxhlet extraction (E2)—revealed distinct phytochemical profiles, with the identification of 60 secondary metabolites, the analytical characteristics of which are detailed in **Table** **8**. This multimodal approach not only confirmed the presence of these compounds but also highlighted the decisive influence of the extraction solvent on the overall chemical composition. A comparative analysis reveals marked variations between the extracts. The decoction (E0) stands out for its richness in phenylethanoids (45.87%), dominated by poliumoside (36.45%) and verbascoside (9.42%), demonstrating the efficiency of aqueous decoction for extracting these polar compounds (**Figure** **6**). The aqueous Soxhlet extract (E1) presents a balanced profile between flavonoids (47.23%) and phenylethanoids (29.84%), while the hydroethanolic Soxhlet extract (E2) shows a clear predominance of nonpolar flavonoids (54.65%) and phenolic acids (21.48%). The differential solubility properties can explain these differences: water favors polar compounds such as glycosylated phenylethanoids, while ethanol exhibits a greater affinity for aglycone flavonoids and less polar phenolic derivatives.

Figure 5HPLC chromatogram of *T. polium* compounds from a) decoction, b) aqueous extract, and c) hydroethanolic extract obtained by Soxhlet.

**Figure:**
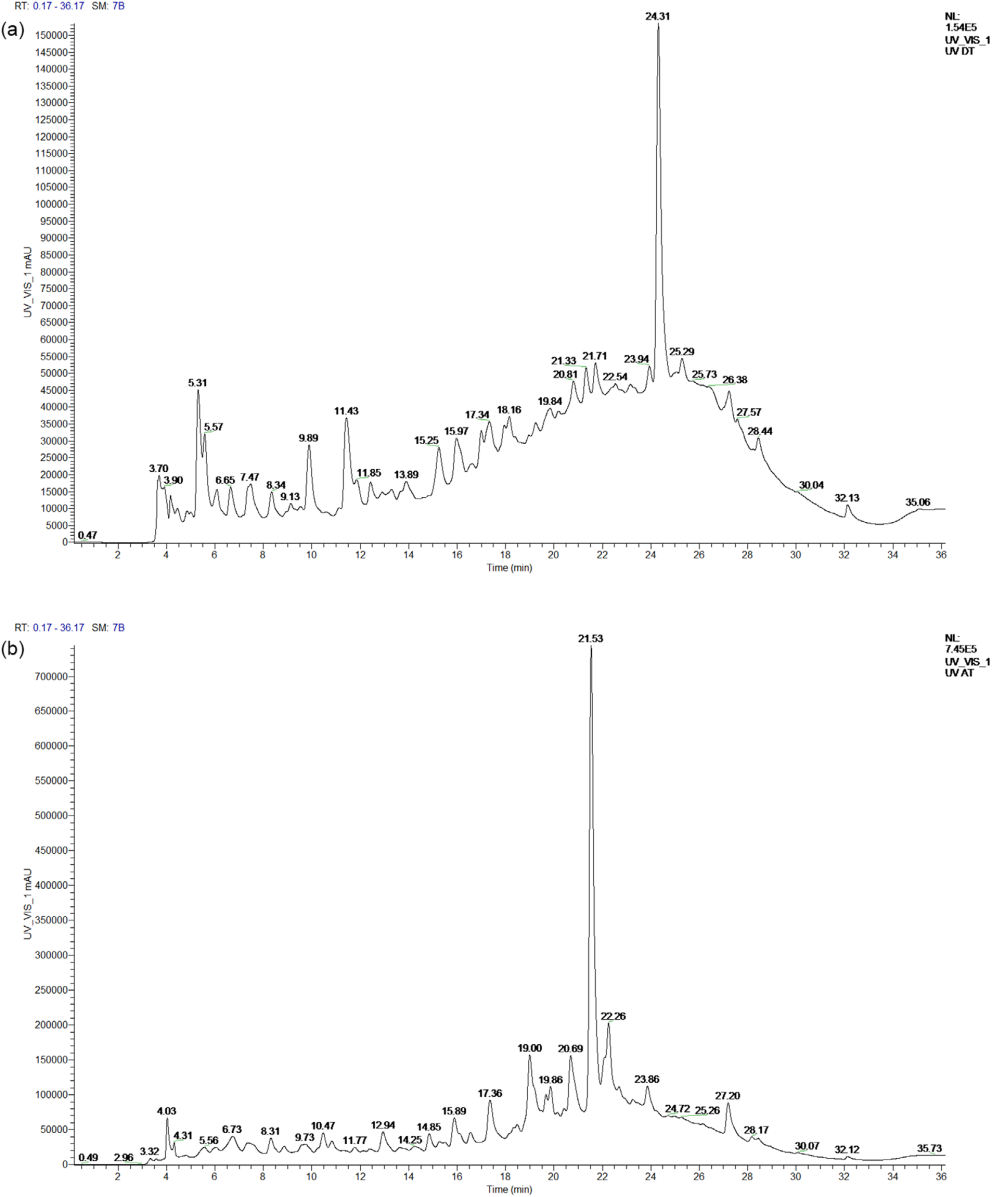

**Figure:**
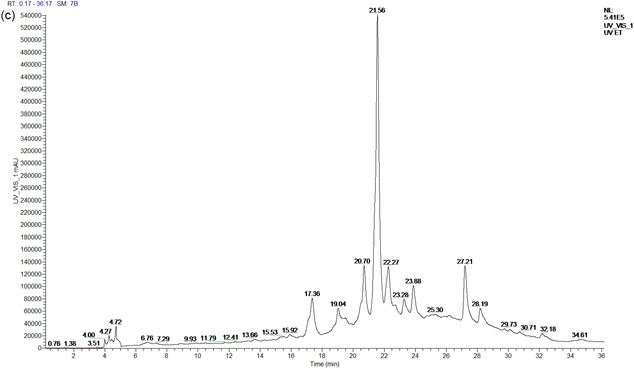

**Table 8:** List of compounds identified in the decoction (E0), aqueous extracts (E1), and ethanolic extracts (E2) of *T. polium* by HPLC/UV‐ESI‐MS.

RT	Molecules	Classes	Exact weights	[M‐H]^−^ [m z^–^^1^]	Fragment Ions [m z^–1^]	Area [%]		
						E (0)	E (1)	E (2)
3.7	p‐Coumaroyl tartaric acid	Phenolic acid	292	291	291‐173‐119	0.00	0.00	3.41
4.03	Myricetin	Flavonoid	318	317	317–179 ‐151	1.49	1.28	5.17
4.31	Pinoresinol	Polyphenol	358	357	357‐151‐137	0.00	0.89	1.71
4.72	Caffeoyl Hexoside	Phenolic acid	342	341	341‐323‐297‐179	1.51	0.00	0.00
5.31	2′‐Hydroxyflavanone	Flavonoid	240	239	239‐135‐107	0.00	0.00	6.43
5.56	Caffeoylmalic acid	Phenolic acid	296	295	295‐179‐133	0.00	0.96	4.79
6.08	Lupulon	Terpenoid	414	413	413‐247‐205	0.00	0.72	2.55
6.65	Kaempferol 3‐malonylglucoside	Flavonoid	534	533	533‐489‐447‐285	1.57	1.95	1.93
7.29	Myricetin‐3‐*O*‐xyloside	Flavonoid	450	449	449‐317‐179	0.56	1.24	0.00
7.47	Oleuropein aglycone	Iridoid	380	379	379‐195‐137	0.00	0.00	2.56
8.31	Glabrol	Flavonoid	392	391	391‐313‐273	0.00	0.68	0.00
8.34	4‐Chlorosalicylic acid	Phenolic acid	172	171	171‐127‐108	0.00	0.00	1.33
8.9	Dimethyl galloyl hexoside	Polyphenol	360	359	359‐197‐169	0.34	0.26	0.00
9.73	(epi)Catechin dihexoside	Flavonoid	614	613	613‐451‐289	0.16	0.31	0.00
9.89	Luteolin 7‐O‐(6″‐malonylglucoside)	Flavonoid	534	533	533‐489‐447‐285	0.00	0.00	3.55
10.48	Oleoside	Iridoid	390	389	389‐227‐195	0.24	1.00	0.00
11.43	Kaempferol	Flavonoid	286	285	285‐257‐153	0.00	0.00	4.60
12.43	Feruloyl‐gamma‐quinide	Phenolic acid	350	349	349‐193‐179	0.00	0.00	0.84
12.94	Chlorogenic acid	Phenolic acid	354	353	353‐191‐179	0.07	0.96	0.00
13.89	Sinapoyl D‐glucoside	Phenolic acid	386	385	385‐223‐193	0.18	0.52	0.73
15.25	Harpagide	Iridoid	364	363	363‐179‐137	0.00	0.00	2.70
15.92	Kaempferol galloyl pentoside	Flavonoid	570	569	569‐441‐285	0.33	1.41	0.00
15.97	1‐Caffeoyl‐beta‐D‐glucose	Phenolic acid	342	341	341‐179‐161	0.00	0.00	2.74
17.36	Naringin	Flavonoid	580	579	579‐271‐151	5.44	2.61	2.85
17.95	2‐O‐caffeoylglucaric acid	Phenolic acid	372	371	371‐179‐193	0.00	1.56	0.76
19	Valoneic acid dilactone	Polyphenol	470	469	469‐313‐211	3.69	6.37	1.42
19.5	Luteolin‐7‐rutinoside	Flavonoid	594	593	593‐285‐217	1.88	2.28	1.01
19.84	Apigenin‐7‐*O*‐glucoside	Flavonoid	432	431	431‐269	0.47	3.54	2.37
20.19	Feruloytartaric acid	Phenolic acid	326	325	325‐193‐149	0.00	0.00	0.97
20.7	Verbascoside	Phenylethanoid	624	623	623‐461‐161	9.42	8.39	3.66
21.53	Poliumoside	Phenylethanoid	770	769	769‐623‐161	36.45	21.45	6.93
22.27	Isorhamnetin‐3‐O‐rutinoside	Flavonoid	624	623	623‐315‐299	8.80	9.68	0.00
22.54	Calcitriol	Vitamin	416	415	415‐397‐379‐363	0.00	0.00	2.31
22.71	Quercitrin	Flavonoid	448	447	447‐301‐179	2.66	4.64	0.00
23.16	Apigenin‐*O*‐glucuronide	Flavonoid	446	445	445‐269‐175	0.00	0.00	0.55
23.26	caffeoyl‐feruloyltartaric acid	Phenolic acid	488	487	487‐193‐179‐149	0.00	4.05	0.00
23.28	3‐O‐coumaroylquinic acid	Phenolic acid	338	337	337‐ 191‐ 163‐ 119	3.65	0.00	0.00
24.31	Apigenin 7‐rutinoside	Flavonoid	578	577	577‐431‐269	5.36	6.61	21.18
24.75	Diosmin (diosmetin‐7‐O‐rutinoside)	Flavonoid	608	607	607‐299‐284	0.62	1.78	0.00
24.96	Apigenin‐4^′^‐*O*‐glucoside	Flavonoid	432	431	431‐341‐269	0.76	1.54	0.00
25.29	Luteolin 7‐diglucuronide	Flavonoid	640	639	639‐285‐175	0.00	0.00	1.72
25.3	Rutin	Flavonoid	610	609	609‐301‐179	1.45	4.34	0.00
26.18	Luteolin	Flavonoid	286	285	285‐241‐197	0.48	3.34	0.00
26.37	Myricetin 3‐glucoside	Flavonoid	480	479	479‐317‐179	0.00	0.00	0.68
27.2	Vanillic acid hexoside	Phenolic acid	330	329	329‐167	5.87	3.80	0.00
27.23	Catechin hydrate	Flavonoid	310	309	309‐245‐179‐125	0.00	0.00	1.45
28.17	Ellagic acid	Polyphenol	302	301	301‐257‐229‐185	0.00	1.83	0.00
28.19	5,7‐dihydroxy‐3,4‐dimetoxyflavone	Flavonoid	314	313	313‐298‐283‐255	2.24	0.00	0.00
28.44	Dihydrosamidine	Flavonoid	388	387	387‐345‐ 315‐ 299	0.00	0.00	1.16
29.73	Apigenin	Flavonoid	270	269	269‐225‐151‐117	0.11	0.00	0.00
30.1	Embelin	Others	294	293	293‐184	0.18	0.00	0.00
30.71	Digallic acid	Polyphenol	322	321	321‐169‐153	0.22	0.00	0.00
32.18	Kaempferol 3‐O‐sinapoyl‐sophoroside 7‐O‐glucoside	Flavonoid	978	977	977‐815‐653‐609‐285	0.40	0.00	0.00
34.61	Hexoside‐O‐galloylquinic acid	Phenolic acid	506	505	343‐331‐191‐ 459‐ 169	0.27	0.00	0.00
34.8	Ellagic acid pentoside	Polyphenol	434	433	301‐257‐229	0.22	0.00	0.00
35.06	Sinapoyl malate	Phenolic acid	340	339	339‐223‐179	0.00	0.00	1.15
37.37	Caffeoyl derivative	Phenolic acid	428	427	427‐373‐339‐297	0.22	0.00	0.00
38.17	Epicatechin gallate	Flavonoid	442	441	441‐289‐169	0.80	0.00	0.00
39.6	Syringic acid hexoside	Phenolic acid	360	359	359‐197‐123	0.00	0.00	4.76
39.88	Caffeic acid‐O‐hexoside	Phenolic acid	342	341	341‐179‐161	1.85	0.00	0.00

**Figure 7:**
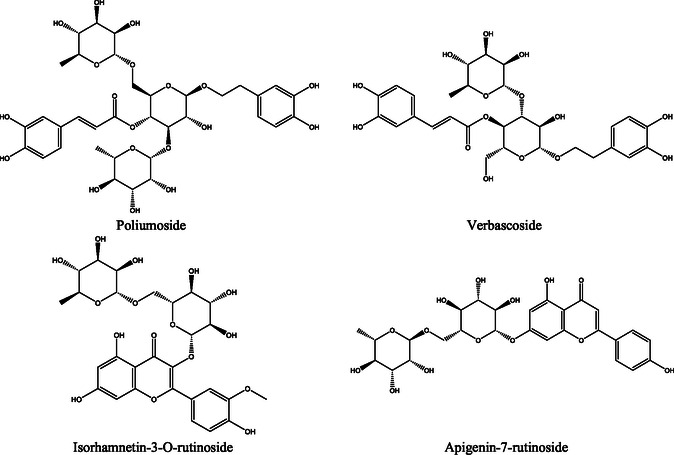
Structure of the main compounds identified in *T. polium* extracts.

The analysis of mass spectra (ESI‐MS) of the major compounds in *T. polium* reveals characteristic fragmentation patterns, enabling precise structural identification. Poliumoside ([M‐H]^−^ m/z 769) exhibits typical fragmentation of glycosylated phenylethanoids, with successive losses of rhamnosyl (m/z 623) and hexose, resulting in the caffeate ion (m/z 161). Verbascoside (m/z 623) shows a loss of the caffeoyl group (m/z 461), followed by the elimination of rhamnose (m/z 315). For flavonoids, apigenin‐7‐rutinoside (m/z 577) fragments via cleavage of the rutinoside group (m/z 269, apigenin aglycone), followed by decarboxylation (m/z 225). Similarly, isorhamnetin‐3‐O‐rutinoside (m/z 623) loses its rutinoside (m/z 315) before undergoing demethylation (m/z 301). Kaempferol (m/z 285) displays the classic fragmentation of flavonols, including retro‐Diels‐Alder cleavage (m/z 257) and the formation of phloroglucinol (m/z 151). Finally, chlorogenic acid (m/z 353) is characterized by the loss of quinic acid (m/z 191) followed by decarboxylation (m/z 179). **Figure** **7** complements this analysis by presenting the chemical structures of the main identified compounds, providing precise structural details.

From a biological perspective, these findings have significant implications. The abundance of phenylethanoids in decoction E(0) implies enhanced protective capabilities, whereas the dominance of flavonoids in aqueous extract E(2) predicts potentially greater antioxidant efficacy. Various molecules demonstrate distinct distributions based on the extract, such as p‐coumaroyl tartaric acid (exclusive to E2) and caffeoyl‐feruloyltartaric acid (exclusive to E1), hence serving as unique chemical markers for each extraction technique. This extensive investigation highlights the crucial importance of selecting the extraction procedure to tailor the phytochemical profile for specific medicinal purposes. The integration of chromatographic and spectrometric analyses provides a comprehensive analysis of T. polium's contents, laying a logical foundation for enhancing extraction techniques for specific applications in phytotherapy and pharmacognosy.

Researchers have examined the phytochemical profile of *T. polium* in the literature, focusing on how various extraction methods impact its phenolic composition. Past research has shown that the polarity of solvents and the extraction technique have a significant effect on the metabolic profile of this species. Goulas et al.^[^ ^103^ ^]^ found that the aqueous decoction of *T. polium* mostly makes polar glycosylated phenylpropanoids like poliumoside and verbascoside. This is because these compounds dissolve well in water. A study by Bahramikia et al.^[^ ^104^ ^]^ also found that hydroalcoholic extraction facilitates the extraction of nonpolar flavonoids, such as apigenin and kaempferol derivatives. The results of this study are in line with those of the previous research, which found that the hydroethanolic extract E(2) had more nonpolar flavonoids (54.65%). Sharififar et al.^[^ ^99^ ^]^ also found that the aqueous extract has a good balance of flavonoids and phenylpropanoids. These results support what we already know: the aqueous extract from Soxhlet E(1) has 47.23% flavonoids and 29.84% phenylethanoids. The presence of certain markers, such as p‐coumaroyl tartaric acid (only found in E2) and caffeoyl‐feruloyltartaric acid (only found in E1), supports the idea that *T. polium* extracts can be different depending on the solvent, as Yasir et al. ^[^ ^105^ ^]^ said before. These results show how important it is to choose the right extraction method based on how you want to use the drug. The aqueous decoction (E0) may be more effective in killing germs and reducing inflammation, while the hydroethanolic extract (E2) may be more effective in fighting cancer and protecting cells from damage. Further in vitro and in vivo studies are needed to confirm these potential effects and refine extraction methods for targeted therapeutic applications.

### 3.8 Study of the Antioxidant Activity of T. polium

We used the DPPH method to test the antioxidant activity of *T. polium* essential oil, and ascorbic acid was used as a positive control. The EO had an IC_50_ value of 5.234 μg mL^–^^1^, which means it has a moderate but significant ability to fight free radicals. Ascorbic acid, on the other hand, had an IC_50_ of 1.540 μg mL^–1^, which shows that it is a better reference antioxidant. The results are similar to what other studies have found about *T. polium*. For example, Hammoudi et al.^[^ ^106^ ^]^ found an IC_50_ of 7.85 μg mL^–1^ for samples taken in Algeria, while Benchikha et al.^[^ ^19^ ^]^ found an IC_50_ of 4.67 μg mL^–1^ for samples taken in Morocco. Other studies have found higher values, such as Maizi et al.^[^ ^107^ ^]^ which found an IC_50_ of 5,500 μg mL^–1^. This suggests that environmental conditions and extraction methods can have a big effect on the results. Also, a study by Ljubuncic et al.^[^ ^17^ ^]^ on Teucrium marum found an IC_50_ of 12.45 μg mL^–1^, while Chedia et al.^[^ ^108^ ^]^ found that Tunisian samples of *T. polium* had weaker activity with an IC_50_ of 15.230 μg mL^–1^. This difference is due to the fact that EO have different chemical makeups, which are affected by genetics and the environment. Thymol, carvacrol, and *α*‐pinene are compounds that are known to have antioxidant properties, and they seem to be connected to *T. polium*'s antioxidant activity.^[^ ^109^ ^]^ There is considerable evidence that the antioxidant activity of EOs doesn’t solely depend on the main compounds, but also on how minor compounds interact with each other.^[^ ^110^ ^]^

**Figure 8:**
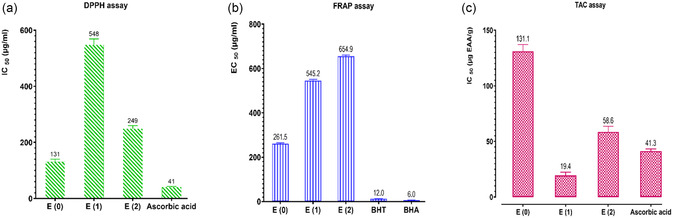
Antioxidant activity of the extracts evaluated by a) the DPPH test, b) the FRAP test, and c) the TAC test. The mean values ± standard deviations of the determinations performed in 3 replicates are reported. The means are significantly different ( *p* < 0.001). Results with different letters are significantly distinct from each other ( *p < *0.001)*.*

**Figure** **8**a presents the total antioxidant capacity of *T. polium* extracts, expressed as IC_50_ (µg AAE/g): E(2) = 41.0, E(1) = 58.6, E(0) = 131.1 µg AAE/g, with ascorbic acid at 19.4. These results indicate a decreasing antioxidant activity in the order: E(2) > E(1) > E(0). The fact that polyphenols and flavonoids are hydrophilic compounds suggests that they are primarily responsible for the antioxidant activity.

These results are in line with what other studies have found in Morocco and other places. For example, a study from Morocco looked at the antioxidant effects of methanolic, ethanolic, and aqueous extracts of a number of plants, including *T. polium*, using the DPPH radical scavenging assay and the FRAP test. The results showed that these extracts have a lot of antioxidant activity because they have a lot of phenolic compounds in them.^[^ ^111^ ^]^

A study of Bakari et al.^[^ ^112^ ^]^ in Tunisia also looked at the polyphenol content and antioxidant activity of methanolic extracts from four plants, one of which was *T. polium*. The results showed that *T. polium* has strong antioxidant activity, which is linked to its high levels of phenolic compounds. The results of these studies back up the idea that aqueous and ethanolic extracts of *T. polium* work best because they can pull out hydrophilic antioxidant compounds. So, it seems that using polar solvents is the best way to get the most antioxidant power out of this medicinal plant.

The DPPH assay results for *T. polium* extracts (Figure 8b) indicate IC_50_ values of ≈131 µg mL^–1^ for E(0), 58.6 µg mL^–1^ for E(1), and 41.0 µg mL^–1^ for E(2). Ascorbic acid exhibits a lower IC_50_ of ≈19.4 µg mL^–1^. Thus, antioxidant activity increases in the order E(0) < E(1) < E(2), with ascorbic acid exhibiting the highest activity (lowest IC_50_).

The ethanolic extract is the one that exhibits the highest antioxidant activity. This efficiency is likely due to ethanol, which enables the extraction of bioactive compounds with varying solubilities, particularly polyphenols and flavonoids. These compounds are well known for their role in neutralizing free radicals due to their hydroxyl groups, capable of donating electrons and stabilizing reactive oxygen species (ROS). A study conducted by Milošević‐Djordjević et al.^[^ ^94^ ^]^ on *T. polium* in Tunisia confirmed that the methanolic extracts of this plant exhibit significant antioxidant activity due to their richness in flavonoids and phenolic acids.

The aqueous extract, although slightly less potent than the ethanolic extract, also shows significant antioxidant activity. Water is a good solvent for extracting certain hydrophilic flavonoids, phenolic acids, and condensed tannins, which are well‐known molecules for their antioxidant effects. These results are consistent with those of Boulanouar, Abdelaziz, and El Atki et al.^[^ ^113^ ^]^ who evaluated the antioxidant activity of the nonvolatile extracts of *T. polium* using the ABTS + •, DPPH•, FRAP, and phosphomolybdenum methods, and highlighted a strong antioxidant activity in the aqueous extracts.

The decocted extract exhibits significantly lower antioxidant activity. This decrease can be attributed to the thermal degradation of certain heat‐sensitive bioactive compounds, particularly some flavonoids and phenolic acids. Stankovic et al. ^[^ ^70^ ^]^ demonstrated that the high temperature applied during decoction can alter the chemical structure of polyphenols, thereby reducing their ability to neutralize free radicals. However, certain thermostable molecules, such as condensed tannins, may help maintain a certain level of antioxidant activity, albeit reduced.

Lastly, BHT and BHA, which are synthetic antioxidants, are much less active than natural extracts of *T. polium*. These results are especially interesting because they support the idea that plant extracts can be a natural alternative to synthetic antioxidants, which some people are against using because they may be toxic in the long term.^[^ ^114^ ^]^ According to the DPPH test, the essential oil of *T. polium* has an IC_50_ of 5.234 µg mL^–1^ and an EC_50_ of 48.19 µg mL^–1^, as determined by the FRAP test.

The FRAP test (Ferric Reducing Antioxidant Power) is a commonly used method to assess the ability of plant extracts to reduce Fe^3+^ ions to Fe^2+^, thereby indicating their antioxidant potential. The results obtained in Figure 8c for the *T. polium* extracts show notable differences depending on the extraction solvent used. The ethanolic extract exhibits the highest reducing activity (0.0586 ± 0.002), followed by the decocted extract (0.1311 ± 0.001), while the aqueous extract shows the lowest activity (0.0194 ± 0.001). Ascorbic acid, used as a standard reference, follows a linear relationship expressed by the Equation 0.0413*x* + 0.0209.

The superior activity of the ethanolic extract can be explained by its ability to efficiently extract polyphenols, flavonoids, and other antioxidant compounds. Alcoholic solvents are well known for their effectiveness in extracting phenolic compounds with high reducing properties.^[^ ^112^ ^]^ A similar study conducted by Kowalczyk et al.^[^ ^115^ ^]^ demonstrated that hydroalcoholic extracts of medicinal plants exhibited higher antioxidant power than aqueous extracts due to the better solubility of flavonoids in mixed water‐alcohol solvents. Additionally, Stanković et al.^[^ ^116^ ^]^ reported that methanolic extracts of *Teucrium montanum*, a species closely related to *T. polium*, also showed strong FRAP activity, confirming the key role of polyphenols in this type of antioxidant activity.

The decocted extract, although less effective than the ethanolic extract, displays notable activity. This can be attributed to the presence of certain thermostable compounds, such as condensed tannins and some phenolic acids resistant to heat.^[^ ^117^ ^]^ However, certain flavonoids and phenolic acids sensitive to high temperatures may be degraded during the decoction process, reducing their overall antioxidant impact.^[^ ^118^ ^]^ A study conducted by Papafotiou et al. (2016) on Mediterranean medicinal plants also highlighted a decrease in antioxidant activity after prolonged thermal treatment, although some more stable polyphenols were preserved.

The aqueous extract exhibits the lowest antioxidant activity, which is expected given the limited solubility of many antioxidant compounds in water alone. Chtourou et al. (2023) demonstrated that aqueous extracts of various plants generally contain fewer polyphenols and flavonoids than extracts obtained with organic or hydroalcoholic solvents. Similarly, Bendif et al. (2018) emphasized that the reducing capacity of an extract is directly linked to its polyphenol content, which explains why aqueous extracts, which typically contain fewer polyphenols, show lower FRAP activity.

Ascorbic acid, used as a reference standard, plays a major role in iron reduction reactions due to its ability to act as an electron donor. However, its effectiveness in FRAP tests can vary depending on experimental conditions, particularly pH and the presence of other antioxidant compounds.^[^ ^109^ ^]^

The findings from this study align with several works conducted on *T. polium* extracts and other medicinal plants with antioxidant potential. Čanadanović‐Brunet et al.^[^ ^119^ ^]^ confirmed that ethanolic extracts of Teucrium montanum displayed high values in FRAP and DPPH tests, highlighting the influence of the solvent type on the extraction of bioactive compounds. Similarly, Vlase et al.^[^ ^120^ ^]^ showed that methanolic extracts of Teucrium chamaedrys exhibited significantly higher FRAP activity than aqueous extracts, due to a higher concentration of phenolic acids and flavonoids.

Comlekcioglu et al. (2022) also did a study on different Mediterranean medicinal plants and found a strong link between the amount of total polyphenols and the FRAP activity. These results show that the chemical makeup of an extract and the way it was extracted have a big effect on how well it works as an antioxidant. Other studies, like those by Farahmandfar et al. (2019), have shown that ethanolic extracts are usually better than aqueous extracts because they can dissolve more types of phenolic compounds.

The three methods DPPH, FRAP, and TAC used to test the antioxidant activity of *T. polium* extracts show that there are big differences depending on the type of extract and the solvent used to get it. The ethanolic extract worked best in all tests, which confirms that it has strong antioxidant properties because it has a lot of polyphenols and flavonoids.^[^ ^58^ ^]^ The decocted extract had moderate activity, probably because it kept some thermostable antioxidants alive, even though heat can break down some bioactive compounds.^[^ ^121^ ^]^ The aqueous extract, on the other hand, had the least activity. This is because many polyphenols don’t dissolve well in water alone.

The differences between the three methods are due to the basic ideas behind each test. The DPPH test, which looks for free radical scavenging, showed that extracts high in flavonoids had a strong ability to act as antioxidants. The FRAP test, which measures how well compounds can reduce other compounds, confirmed this trend by showing how polyphenols affect the change of Fe^3+^ ions to Fe^2+^. Lastly, the TAC test showed that the extracts could neutralize a wide range of reactive oxygen species. This supports the idea that *T. polium* could be a natural source of antioxidants that could be used in medicine and the food industry.^[^ ^31^ ^]^

These results back up what other studies have found about the antioxidant properties of Mediterranean medicinal plants. They show that the choice of extraction solvent is very important for getting the most antioxidant activity out of the extracts. So, using hydroethanolic or methanolic extracts might be better for making *T. polium* more useful as a medicine.

### 3.9 Study of the Antimicrobial Activity

The test of the antimicrobial activity of *T. polium* EO showed that it worked better against *Staphylococcus aureus* (18.4 ± 1.2 mm) and *C. albicans* (20.1 ± 1.5 mm), but it was less effective against *K. pneumoniae* (11.3 ± 1.1 mm) and *S. cerevisiae* (10.5 ± 0.8 mm). Carvacrol (31.5%), thymol (18.7%), *β*‐caryophyllene (12.3%), and *α*‐pinene (8.9%) are the main compounds that seem to be responsible for this activity.

Carvacrol and thymol are more effective against *S. aureus* and *C. albicans* because they can break down cell membranes, which lets intracellular components leak out.^[^ ^122^ ^]^ These monoterpenic phenols are known to kill bacteria and fungi by making cell membranes less flexible and stopping enzymes that microorganisms need to live. On the other hand, Gram‐negative bacteria like *E. coli* and *K. pneumoniae*, whose outer membrane makes it hard for lipophilic compounds to get through, were more resistant. The moderate effect on Aspergillus niger and *S. cerevisiae* may be because these fungi can make defenses against oxidative stresses caused by essential oils.

These results are consistent with previous studies showing that EO rich in carvacrol and thymol possess strong antimicrobial activity, particularly against Gram‐positive bacteria and pathogenic yeasts. *β*‐caryophyllene and *α*‐pinene, although less active individually, could play a synergistic role by facilitating the penetration of phenols into microbial membranes.^[^ ^98^ ^]^ Thus, the EO of *T. polium* presents promising potential as a natural antimicrobial agent, particularly against infections caused by S. aureus and C. albicans, which justifies further research into its clinical applications.

The results in **Table** **9** show that the extracts of *T. polium*, prepared from a stock solution of 30 mg mL^–^^1^, exhibit significant antibacterial activity against several microbial strains. Most of the extracts display MBC/MIC (minimum bactericidal concentration/minimum inhibitory concentration) ratios below 2, indicating bactericidal activity. However, an exception is observed with Saccharomyces cerevisiae, where the aqueous extract shows an MBC/MIC ratio >2, suggesting a bacteriostatic rather than bactericidal action. This difference may be related to the hydrophilic nature of the active compounds present in the aqueous extract, which might be less effective against certain yeasts.^[^ ^22^ ^]^

**Table 9:** Antimicrobial activity of *T. polium* extracts: MIC, MBC/MFC, and killing index (MBC/MIC or MFC/MIC) in mg/mL.

*Micro‐organisms*		E(0)	E(1)	E(2)
*Escherichia coli*	MIC	–	–	0.576
	MBC	–	–	0.81
	MBC/MIC	–	–	1.40
*Staphylococcus aureus*	MIC	0.6125	0.524	0.290
	MBC	0.600	0.432	0.100
	MBC/MIC	0.979	0.797	0.344
*Klebsiella pneumoniae*	MIC	0.597	–	0.456
	MBC	0.657	–	0.431
	MBC/MIC	1.10	–	0.94
*Saccharomyces cerevisiae*	MIC	–	0.524	0.609
	MBC	–	1.09	0.861
	MBC/MIC	–	2.08	1.41
*Aspergillus niger*	MIC	–	–	0.453
	MFC	–	–	0.123
	MFC/MIC	–	–	0.27
*Candida albicans*	MIC	0.440	0.325	0.201
	MFC	0.279	0.303	0.169
	MFC/MIC	0.63	0.93	0.84

*(–) : not determined.

*T. polium*'s antibacterial effect is mostly due to its high levels of secondary metabolites like flavonoids, terpenoids, and tannins, which are known to kill bacteria.^[^ ^59^ ^]^ A study done in Iran showed that ethanolic and methanolic extracts of *T. polium*, made from stock solutions of 200–500 and 400–600 mg mL^–^^1^, respectively, are very effective against Staphylococcus aureus and *E. coli*.^[^ ^59^ ^]^ These results support other research that says ethanolic and methanolic extracts work better than aqueous extracts because they can get more different bioactive compounds out of plants.^[^ ^98^ ^]^

Researchers found that *T. polium* extracts worked better on Gram‐positive bacteria than on Gram‐negative bacteria. The main reason for this difference is that the structure of the bacterial cell wall is different. It is true that Gram‐negative bacteria have an outer membrane that is full of lipopolysaccharides (LPS). This membrane protects the bacteria by keeping antimicrobial compounds from getting inside.^[^ ^123^ ^]^ Gram‐positive bacteria, on the other hand, do not have this outer membrane, so they are more likely to be harmed by the polyphenols and terpenoids in the extracts.^[^ ^124^ ^]^

The type of bioactive compounds is just as important as the structure of the membrane when it comes to how well they kill microbes. For example, flavonoids and terpenes in *T. polium* can bind to the membrane proteins of Gram‐positive bacteria, which makes them unstable and causes them to break down.^[^ ^125^ ^]^ The strength of the antibacterial activity that was seen also depends on experimental factors like the concentration of the extracts, the size of the application discs in diffusion tests, and the genetic makeup of the bacterial strains.^[^ ^125^ ^]^

*T. polium* has antimicrobial properties that are similar to those of other medicinal plants that are high in phenolic compounds. For instance, oregano (Origanum vulgare) has carvacrol and thymol, which work against *S. aureus* and *P. aeruginosa* in the same way.^[^ ^125^ ^]^

### 3.10 Acute Toxicity

The results of this short‐term toxicity test show that none of the extracts are toxic, even at a dose of 2 g kg^–^^1^. There were no signs of toxicity (like diarrhea, vomiting, and problems with movement) or death during the monitoring period.

### 3.11 Study of the Antidiabetic Activity In Vitro

We tested the EO of *T. polium* to see if it could help with diabetes in both living and dead cells. The EO significantly stopped *α*‐glucosidase (73 ± 2.1% at 0.2 mg mL^–^^1^) and *α*‐amylase (65 ± 1.8% at 0.2 mg mL^–1^) from breaking down carbohydrates into glucose in vitro. This inhibition is similar to that of acarbose, a common enzyme inhibitor, which means it could lower blood sugar levels by slowing down the absorption of glucose in the intestines. Previous research has shown that *T. polium* extracts lower blood sugar levels by increasing insulin secretion and the expression of glucose transporters (GLUT4) in peripheral tissues.^[^ ^126^ ^]^ This supports the idea that *T. polium* has antidiabetic properties. Also, the presence of *β*‐caryophyllene in the EO could make these effects stronger by activating type 2 cannabinoid receptors (CB2), which help control how glucose and lipids are broken down and used by the body.^[^ ^85^ ^]^

The results shown in **Table** **10** show that *T. polium* extracts have a strong effect on *α*‐amylase and *α*‐glucosidase enzymes, which are two important enzymes that help control blood sugar levels. The aqueous extract worked best against *α*‐amylase, with an IC_50_ of 0.42 mg mL^–1^. The ethanolic extract worked best against *α*‐glucosidase, with an IC_50_ of 0.20 mg mL^–1^. Acarbose, a standard inhibitor, had IC_50_ values of 0.59 mg mL^–1^ for *α*‐amylase and 0.38 mg mL^–1^ for *α*‐glucosidase, which is a lot lower than the other two. These findings suggest that *T. polium* might be a natural or complementary option to standard treatments for type 2 diabetes.

**Table 10:** Effect of *T. polium* extracts on *α*‐amylase and *α*‐glucosidase activities.

Extract	IC_50_ [mg mL^–1^]	
	*α*‐Amylase	*α*‐Glucosidase
Decocted extract	0.62 ± 0.22	0.33 ± 0.45
Aqueous extract	0.42 ± 0.10	0.28 ± 0.78
Ethanolic extract	0.51 ± 0.09	0.20 ± 0.11
Acarbose (control)	0.59 ± 0.23	0.38 ± 0.15

The values obtained in this study are generally consistent with previous research. For example, Santos‐Buelga et al.^[^ ^127^ ^]^ reported an IC_50_ of 111.68 µg mL^–1^ (0.111 mg mL^–1^) for *α*‐amylase inhibition by a hydroalcoholic extract of T. polium, indicating higher activity than what was found. Another study revealed IC_50_ values of 0.55 mg mL^–1^ for *α*‐amylase and 0.28 mg mL^–1^ for *α*‐glucosidase, which are close to the values obtained with the aqueous extract and decoction in our study.^[^ ^128^ ^]^ These differences can be attributed to variations in extraction methods, experimental conditions, and enzymatic sources used.

Acarbose, a drug commonly used in diabetes management, exhibits variable IC_50_ values depending on the study. For instance, some research reports IC_50_ values ranging from 0.110 to 52.2 µg mL^–1^ for *α*‐amylase inhibition and 28.8 µg mL^–1^ for *α*‐glucosidase. These variations reflect differences in experimental models used. In our study, *T. polium* extracts demonstrated inhibitory activity similar to or even superior to acarbose on *α*‐glucosidase, highlighting their potential as natural hypoglycemic agents.

From a biological perspective, these results confirm that *T. polium* contains bioactive metabolites capable of inhibiting carbohydrate‐digesting enzymes, thus contributing to the reduction of glucose absorption. The superior activity of the ethanolic extract on *α*‐glucosidase suggests that the compounds responsible for this inhibition are more soluble in ethanol, possibly flavonoids or polyphenols.^[^ ^128^ ^]^ These findings support the traditional use of *T. polium* in diabetes management and justify further studies to identify the active molecules and evaluate their safety and efficacy in vivo.

### 3.12 Study of the Antidiabetic Activity In Vivo

The oral administration of EO (70 mg/kg/day) to streptozotocin (STZ)‐induced diabetic rats resulted in a significant reduction in fasting blood glucose levels (8.5 ± 0.6 mmol L^–1^ after 14 days, compared to 15.2 ± 1.1 mmol L^–1^ before treatment, *p < *0.05). Additionally, the essential oil improved glucose tolerance. These effects could be attributed to phenolic monoterpenes, primarily carvacrol and thymol, which have demonstrated hypoglycemic effects in previous studies.^[^ ^129^ ^]^

The results in **Table** **11** show a significant change in fasting blood glucose levels in diabetic rats treated with *T. polium* extracts. After 30 days of treatment, the blood glucose levels of untreated diabetic rats continued to increase (2.56 → 3.42 → 3.79 g L^–^^1^), confirming the worsening of hyperglycemia in the absence of treatment. In contrast, the groups treated with *T. polium* extracts showed a progressive decrease in blood glucose levels, with the aqueous extract and ethanolic extract reducing glucose levels to values close to those of the group treated with glibenclamide (1.18 and 1.20 g L^–^^1^, respectively, compared to 1.10 g L^–^^1^ for glibenclamide).

**Table 11:** Effect of the decocted extract of *T. polium* on fasting blood glucose in STZ‐induced diabetic mice. Data are expressed as mean (with ± SD/SEM ; *n* = 6 ; *p < *0.05).

Fasting blood glucose levels [g L^–^^1^]			
Groups	7th Day	14th Day	30th Day
Untreated normal	0.98 ± 0.08	1.10 ± 0.09	1.07 ± 0.11
Normal treated with the extract	0.88 ± 0.07	1.01 ± 0.08	0.90 ± 0.07
Untreated diabetic	2.56 ± 0.30	3.42 ± 0.39	3.79 ± 0.48
Diabetic treated with the decocted extract	2.11 ± 0.28	1.40 ± 0.20	1.25 ± 0.18
Diabetic treated with the aqueous extract	1.65 ± 0.22	1.22 ± 0.16	1.18 ± 0.15
Diabetic treated with the hydro‐ethanolic extract	1.78 ± 0.24	1.26 ± 0.18	1.20 ± 0.17
Diabetic treated with glibenclamide	1.23 ± 0.11	1.17 ± 0.11	1.10 ± 0.11

These results are consistent with several previous studies. İşcan et al. ^[^ ^130^ ^]^ observed a significant reduction in blood glucose levels in diabetic rats treated with an aqueous extract of T. polium, attributing this effect to an improvement in peripheral glucose metabolism rather than a direct increase in insulin secretion. Albadr et al. (2022) also reported a significant decrease in blood glucose levels after administration of the extract, suggesting stimulation of insulin secretion by pancreatic *β*‐cells.

The aqueous extract was found to be the most effective in reducing blood glucose levels (1.65 → 1.22 → 1.18 g L^–^^1^), followed by the ethanolic extract (1.78 → 1.26 → 1.20 g L^–^^1^) and the decoction (2.11 → 1.40 → 1.25 g L^–^^1^). The greater efficacy of the aqueous extract could be related to better preservation of water‐soluble active compounds, notably flavonoids and polyphenols, which are known for their antioxidant and hypoglycemic effects.^[^ ^131^ ^]^

The study by İşcan et al.^[^ ^130^ ^]^ showed that ethanolic extracts of *T. polium* possess inhibitory properties against *α*‐glucosidase and *α*‐amylase, which can slow down carbohydrate digestion and reduce glucose absorption. This effect could explain why the ethanolic extract produced slightly lower results than the aqueous extract but still close to those of glibenclamide.

The group treated with glibenclamide showed a rapid and stable reduction in blood glucose levels (1.23 → 1.17 → 1.10 g L^–^^1^), confirming its well‐established efficacy as a hypoglycemic agent. The *T. polium* extracts, particularly the aqueous extract, produced comparable results to glibenclamide after 30 days of treatment, suggesting significant therapeutic potential. However, unlike glibenclamide, which primarily acts by stimulating insulin secretion, *T. polium* extracts may exert a combined effect on insulin sensitivity and the modulation of digestive enzymes.^[^ ^8^ ^]^

These results confirm the promising hypoglycemic effect of *T. polium*, in agreement with several previous studies. The aqueous extract appears to be the most effective, likely due to its high concentration of water‐soluble flavonoids and polyphenols. However, while the effects are comparable to those of glibenclamide, further studies, including clinical trials, are necessary to confirm the efficacy and safety of *T. polium* in humans.

### 3.13 Biochemical Parameters (After 30 Days of Treatment)

After 30 days of treatment with *T. polium* EO (50 mg/kg/day), a significant improvement (*p < *0.01) in biochemical parameters was observed in diabetic rats (**Table** **12**).

**Table 12:** Effects of *T. polium* essential oil on biochemical parameters (after 30 days of treatment). Data are expressed as mean (with ± SD/SEM; *n* = 3; *p < *0.01).

Parameters	Untreated diabetics	Diabetics treated with EO
Total proteins	54.2 ± 3.4	62.8 ± 4.1
Urea [mg dL^–1^]	42.5 ± 3.2	31.7 ± 2.8
Cholesterol [g L^–1^]	2.15 ± 0.18	1.45 ± 0.12
Creatinine [mg L^–1^]	9.2 ± 0.8	6.7 ± 0.6
Triglycerides [g L^–1^]	1.85 ± 0.12	1.09 ± 0.08
Uric Acid [mg L^–1^]	58.4 ± 4.1	45.2 ± 3.6
ASAT [UI L^–1^]	96.3 ± 7.2	72.8 ± 5.4
ALAT [UI L^–1^]	78.5 ± 6.8	55.9 ± 4.3

The total protein level increased from 54.2 ± 3.5 g L^–^^1^ (in the untreated diabetic group) to 62.8 ± 4.1 g L^–1^ after treatment. This improvement suggests better regulation of protein metabolism and an overall enhancement of the animals’ general condition. At the same time, urea and creatinine levels, which are indicators of kidney function, decreased from 42.5 ± 3.2 to 31.7 ± 2.8 mg dL^–1^ and from 9.2 ± 0.8 to 6.7 ± 0.6mg L^–^^1^, respectively. This reduction indicates a potential protective effect of the EO against renal complications often associated with diabetes.

The total cholesterol level decreased from 2.15 ± 0.18 to 1.45 ± 0.12g L^–^^1^, representing a 32.5% reduction (*p < *0.01), indicating a significant hypolipidemic effect.

Similarly, triglycerides, which are often elevated in diabetic patients, were significantly reduced from 1.85 ± 0.12 to 1.09 ± 0.08 g L^–^^1^ (*p < *0.01), suggesting better control of lipid metabolism and a potential cardioprotective effect. Uric acid, a marker associated with oxidative stress and metabolic complications, also decreased from 58.4 ± 4.1 to 45.2 ± 3.6 mg L^–^^1^, supporting the hypothesis that the EO has an antioxidant action.

The liver enzymes ASAT and ALAT, which indicate liver damage, significantly decreased after treatment. The ASAT level dropped from 96.3 ± 7.2 to 72.8 ± 5.4 IU L^–^^1^, while ALAT decreased from 78.5 ± 6.8 to 55.9 ± 4.3  IU L^–1^. These results suggest that *T. polium* essential oil may have a protective effect against diabetes‐induced hepatotoxicity.

**Figure 9:**
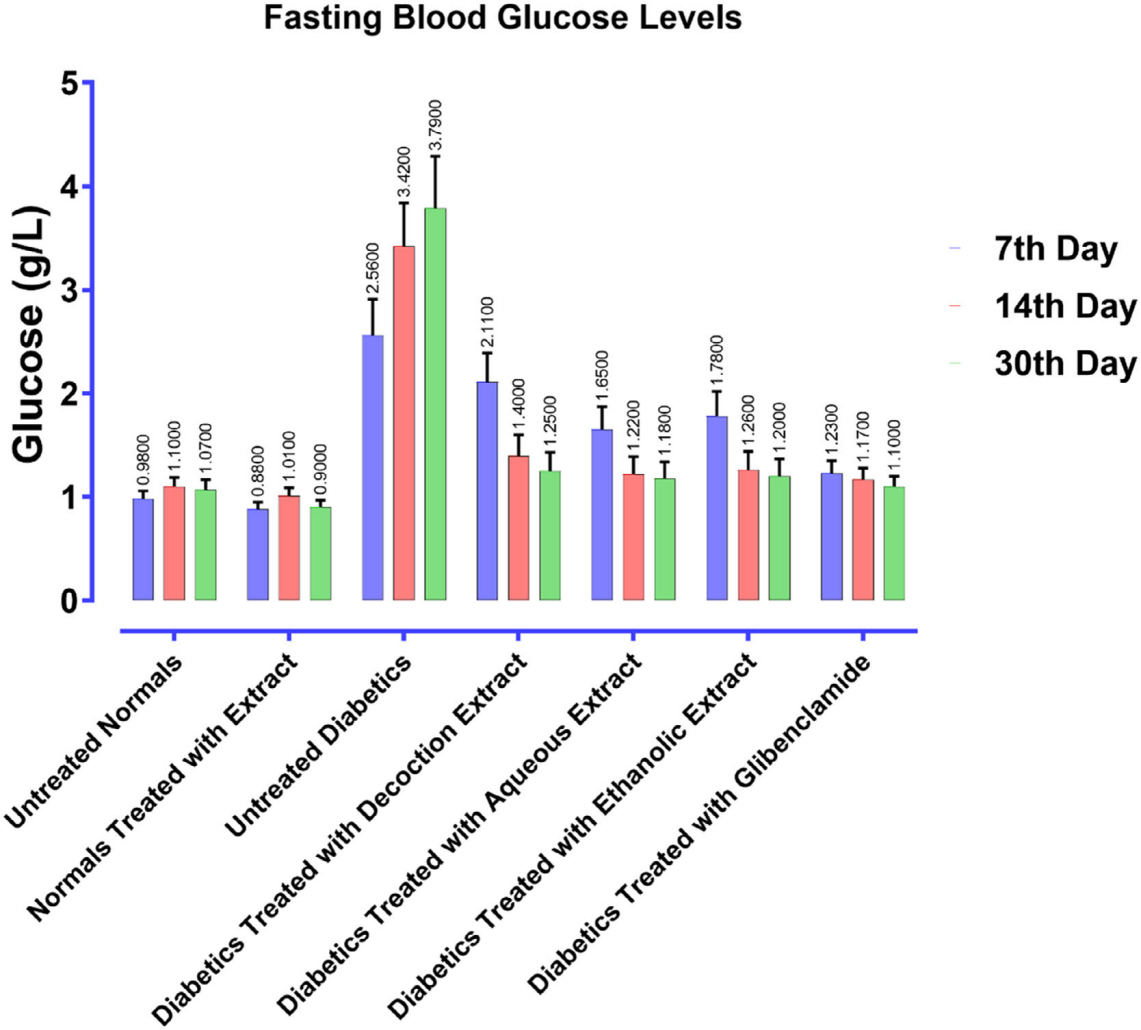
Effects of *T. polium* extracts on biochemical parameters (after 30 days of treatment). Data are expressed as mean (with ± SD/SEM; *n* = 6; *p <* 0.05).

The results in **Table** **13** and **Figure** **9** present biochemical parameters in untreated normal rats and after treatment with three types of *T. polium* extracts (decocted [E0], aqueous [E1], and ethanolic [E2]): untreated normal rats showed an average value of 63.7 ± 2.1 g L^–^^1^. After treatment, a slight increase in total proteins was observed in all treated groups (E0: 68.2 ± 3.6 g L^–^^1^, E1: 67.0 ± 2.1 g L^–^^1^, E2: 67.2 ± 3.7 g L^–^^1^). This increase suggests that the administration of the extracts could improve nutritional status or promote plasma protein synthesis, possibly due to the stimulating action of the bioactive compounds present in the plant.

**Table 13:** Effects of *T. polium* extracts on biochemical parameters (after 30 days of treatment). Data are expressed as mean (with ± SD/SEM; *n* = 3; *p < *0.01).

	Untreated normal	Treated with decoction extract (E0)	Treated with aqueous extract (E1)	Treated with ethanolic extract (E2)
Total proteins	63.7 ± 2.1	68.2 ± 3.6	67.0 ± 2.1	67.2 ± 3.7
Urea [mg dL^–1^]	31.2 ± 2.6	32.4 ± 2.9	29.3 ± 2.6	29.8 ± 2.1
Cholesterol [g L^–1^]	0.74 ± 0.09	0.65 ± 0.03	0.61 ± 0.03	0.63 ± 0.03
Creatinine [mg L^–1^]	6.5 ± 0.6	6.4 ± 0.6	6.1 ± 0.2	6.2 ± 0.5
Triglycerides [g L^–1^]	0.72 ± 0.08	0.63 ± 0.06	0.59 ± 0.04	0.61 ± 0.08
Uric acid [mg L^–1^]	3.5 ± 0.1	3.2 ± 0.3	3.0 ± 0.2	3.1 ± 0.3
AST [UI L^–1^]	45.6 ± 4.0	42.8 ± 3.9	40.1 ± 3.5	41.5 ± 3.7
ALT [UI L^–^^1^]	38.6 ± 3.7	35.2 ± 3.1	34.8 ± 3.0	35.0 ± 3.4

The control group displayed a urea value of 31.2 ± 2.6 mg dL^–^^1^. Treatment with the decocted extract (E0) showed a slight increase to 32.4 ± 2.9 mg dL^–1^, while treatments with aqueous (E1) and ethanolic (E2) extracts induced a significant decrease (29.3 ± 2.6 and 29.8 ± 2.1 mg dL^–1^, respectively). The reduction in urea levels in the E1 and E2 groups could be interpreted as an improvement in kidney function or a reduction in protein degradation, consistent with studies highlighting the nephroprotective effects of polyphenol‐rich extracts.^[^ ^132^ ^]^

In control rats, cholesterol levels were 0.74 ± 0.09 g L^–^^1^. After treatment, a progressive decrease in levels was observed: 0.65 ± 0.03 g L^–^^1^ with the decoction extract (E0), 0.61 ± 0.03 g L^–^^1^ with the aqueous extract (E1), and 0.63 ± 0.03 g L^–^^1^ with the ethanolic extract (E2). This reduction suggests a hypolipidemic effect of *T. polium* extracts, possibly due to the presence of flavonoids and other bioactive compounds that enhance lipid metabolism.^[^ ^133^ ^]^

Creatinine levels remain relatively stable with a slight downward trend in the treated groups (untreated normal: ≈6.5 mg L^–^^1^, E0: 6.4 ± 0.57 mg L^–1^, E1: 6.1 ± 0.2 mg L^–1^, E2: 6.2 ± 0.5 mg L^–1^). This decrease could indicate a marginal improvement in kidney function or a reduction in metabolic stress induced by the treatment, consistent with observations from previous studies on the protective effects of plant extracts.^[^ ^72^ ^]^

Triglyceride levels decrease from 0.72 ± 0.08 g L^–^^1^ in the control group to 0.63 ± 0.06 g L^–1^ (E0), 0.59 ± 0.04 g L^–1^ (E1), and 0.61 ± 0.08 g L^–1^ (E2). This reduction is a positive indicator of an improved lipid profile, reinforcing the hypothesis of a hypolipidemic effect of the extracts, particularly through the action of antioxidants present in T. polium, which promote better regulation of lipid metabolism.^[^ ^133^ ^]^

Uric acid levels are reduced in all treated groups (E0: 3.2 ± 0.3 mg L^–1^, E1: 3.0 ± 0.2 mg L^–1^, E2: 3.1 ± 0.3 mg L^–1^) compared to the control group (3.5 ± 0.1 mg L^–1^). This decrease suggests an improvement in purine metabolism or facilitated renal excretion, potentially linked to the antioxidant properties of the extracts, which reduce oxidative stress and consequently the production of uric acid.^[^ ^72^ ^]^

The liver enzymes ASAT and ALAT are markers of liver function. In the control group, the values are 45.6 ± 4.0 UI L^–^^1^ for ASAT and 38.6 ± 3.7 UI L^–1^ for ALAT. In treated rats, these values decrease. The reduction in ASAT and ALAT levels indicates a hepatoprotective effect of *T. polium* extracts, which could protect the liver against oxidative damage and improve liver function, as suggested by several studies.^[^ ^133^ ^]^

The administration of different *T. polium* extracts in normal rats induces beneficial changes in various biochemical parameters. The extracts, particularly in aqueous (E1) and ethanolic (E2) forms, improve the lipid profile, reduce markers of renal stress (urea and creatinine), and enhance liver functions (reduction in ASAT and ALAT). These results align with existing literature highlighting the antioxidant, hypolipidemic, and hepatoprotective properties of *T. polium*.^[^ ^19^ ^]^

## 4 Conclusions

This research emphasizes the antidiabetic, antioxidant, and antimicrobial properties of *Teucrium polium*. The extracts and essential oil, which are rich in phenylpropanoids and flavonoids (such as verbascoside, poliumoside, and apigenin‐7‐rutinoside) as well as key monoterpenes (including carvacrol, thymol, and *γ*‐terpinene), demonstrate mechanisms that align with enhanced glucose homeostasis and defense against oxidative stress and pathogens. Our findings indicate that carbohydrate‐digesting enzyme inhibition and measurable antioxidant action contribute to improved postprandial glycaemic response, as evidenced by in vivo signals. Additional research is required to validate long‐term safety, delineate pharmacokinetics, and ascertain clinical efficacy. Pending these validations, T. polium may serve as a potential adjuvant in the management of type 2 diabetes.

## Conflict of Interest

The authors declare no conflict of interest.

## Author Contributions

**Hajar El Ouadni**: conceptualization, methodology, software, investigation, writing—original draft preparation, writing—review and editing. **Aziz Drioiche**: conceptualization, methodology, software, writing—original draft preparation, writing—review and editing. **Fadoua El Makhoukhi**: methodology, formal analysis, supervision. **Omkulthom Al Kamaly**: methodology, formal analysis, funding acquisition. **Hannou Zerkani**: investigation. **Smail Amalich**: investigation. **Imane Tagnaout**: investigation. **Mohamed Radi**: investigation. **Yahya Cherrah**: methodology, writing—review and editing, supervision. **Touriya Zair**: methodology, writing—review and editing, supervision. **Katim Alaoui**: validation, writing—original draft preparation, visualization, supervision, project administration.

## Data Availability

The data that support the findings of this study are available from the corresponding author upon reasonable request.
